# Supplementary material for: Sex-specific KDM6A-HNF4A-CREBH network controls lipoprotein cholesterol metabolism and atherosclerosis via epigenetic reprograming of hepatocytes
Source: Nat Commun. 2026 Mar 23;17:3945. doi: 10.1038/s41467-026-70846-w (PMC13133126; doi:10.1038/s41467-026-70846-w)
Supplement: Supplementary file 1 — Supplementary Information [file 41467_2026_70846_MOESM1_ESM.pdf]

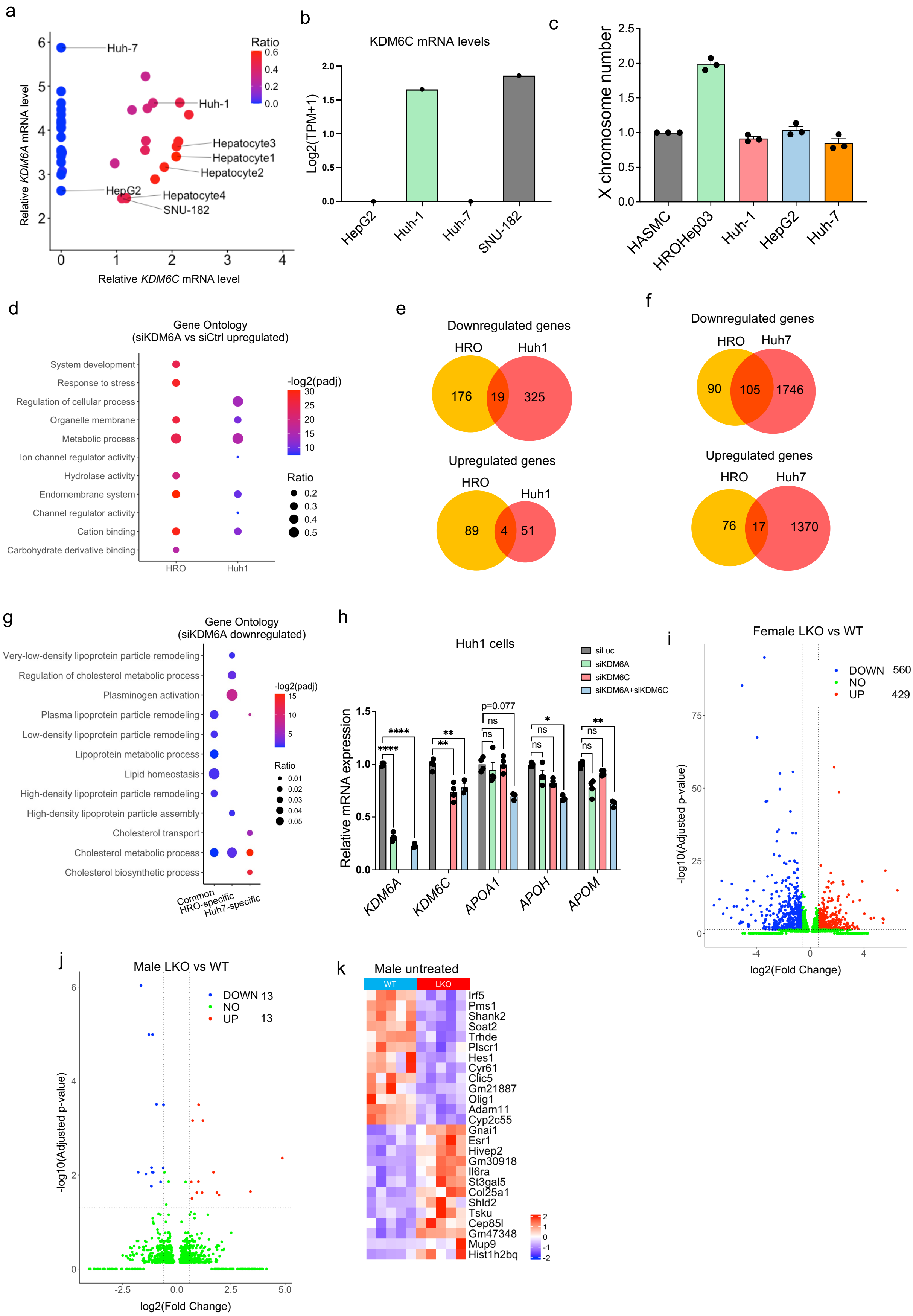
a
Relative KDM6A mRNA level
Relative KDM6C mRNA level
Ratio
Huh-7
Huh-1
Hepatocyte3
Hepatocyte1
Hepatocyte2
HepG2
Hepatocyte4
SNU-182
b
KDM6C mRNA levels
Log2(TPM+1)
HepG2
Huh-1
Huh-7
SNU-182
c
X chromosome number
HASMC
HROHep03
Huh-1
HepG2
Huh-7
d
Gene Ontology
(siKDM6A vs siCtrl upregulated)
System development
Response to stress
Regulation of cellular process
Organelle membrane
Metabolic process
Ion channel regulator activity
Hydrolase activity
Endomembrane system
Channel regulator activity
Cation binding
Carbohydrate derivative binding
HRO
Huh1
-log2(padj)
Ratio
e
Downregulated genes
HRO
Huh1
176
19
325
Upregulated genes
HRO
Huh1
89
4
51
f
Downregulated genes
HRO
Huh7
90
105
1746
Upregulated genes
HRO
Huh7
76
17
1370
g
Gene Ontology
(siKDM6A downregulated)
Very-low-density lipoprotein particle remodeling
Regulation of cholesterol metabolic process
Plasminogen activation
Plasma lipoprotein particle remodeling
Low-density lipoprotein particle remodeling
Lipoprotein metabolic process
Lipid homeostasis
High-density lipoprotein particle remodeling
High-density lipoprotein particle assembly
Cholesterol transport
Cholesterol metabolic process
Cholesterol biosynthetic process
Common
HRO-specific
Huh7-specific
-log2(padj)
Ratio
h
Huh1 cells
siLuc
siKDM6A
siKDM6C
siKDM6A+siKDM6C
Relative mRNA expression
p=0.077
KDM6A
KDM6C
APOA1
APOH
APOM
i
Female LKO vs WT
DOWN 560
NO
UP 429
-log10(Adjusted p-value)
log2(Fold Change)
j
Male LKO vs WT
DOWN 13
NO
UP 13
-log10(Adjusted p-value)
log2(Fold Change)
k
Male untreated
WT
LKO
Irf5
Pms1
Shank2
Soat2
Trhde
Plscr1
Hes1
Cyr61
Clic5
Gm21887
Olig1
Adam11
Cyp2c55
Gnai1
Esr1
Hivep2
Gm30918
Il6ra
St3gal5
Col25a1
Shld2
Tsku
Cep85l
Gm47348
Mup9
Hist1h2bq

Supplementary Figure 1: **(a)** Correlation of *KDM6A* mRNA expression with *KDM6C* changes in 31 male liver cell lines and 4 male patients. The color represents the *KDM6A/KDM6C* ratio in each cell line. **(b)** *KDM6C* mRNA levels in different types of male liver cell lines. **(c)** X chromosome number in different types of male liver cell lines. **(d)** Gene ontology analysis in the increased genes upon KDM6A knockdown in HRO and Huh1 cells. The size of the dots shows the gene ratio; the color of each dot represents -log2(padj) of each pathway. **(e)** Venn diagram of the downregulated (upper panel) and upregulated (lower panel) genes between HRO and Huh1 cells upon KDM6A knockdown. **(f)** Venn diagram of the downregulated (upper panel) and upregulated (lower panel) genes between HRO and Huh7 cells upon KDM6A knockdown. **(g)** Gene ontology analysis of HRO- and Huh7-specific and commonly regulated genes. The size of the dots represents gene ratio, and the color of the dots shows -log2padj. **(h)** qPCR analysis of key lipoprotein genes in siKDM6A, siKDM6C and double knockdown Huh1 cells. (n=3 or 4 in each group), one-way ANOVA followed by Tukey's test. Volcano plot showing the transcriptomic changes upon hepatocyte-specific KDM6A knockout in **(i)** female and **(j)** male mice. The blue, red and green dots represent downregulated, upregulated and unchanged genes. **(k)** Heatmap showing transcriptomic changes upon KDM6A liver knockout in male chow diet mice. All data are represented as mean ± s.e.m.*$P < 0.05$, **$P < 0.01$, ***$P < 0.001$, ****$P < 0.0001$.

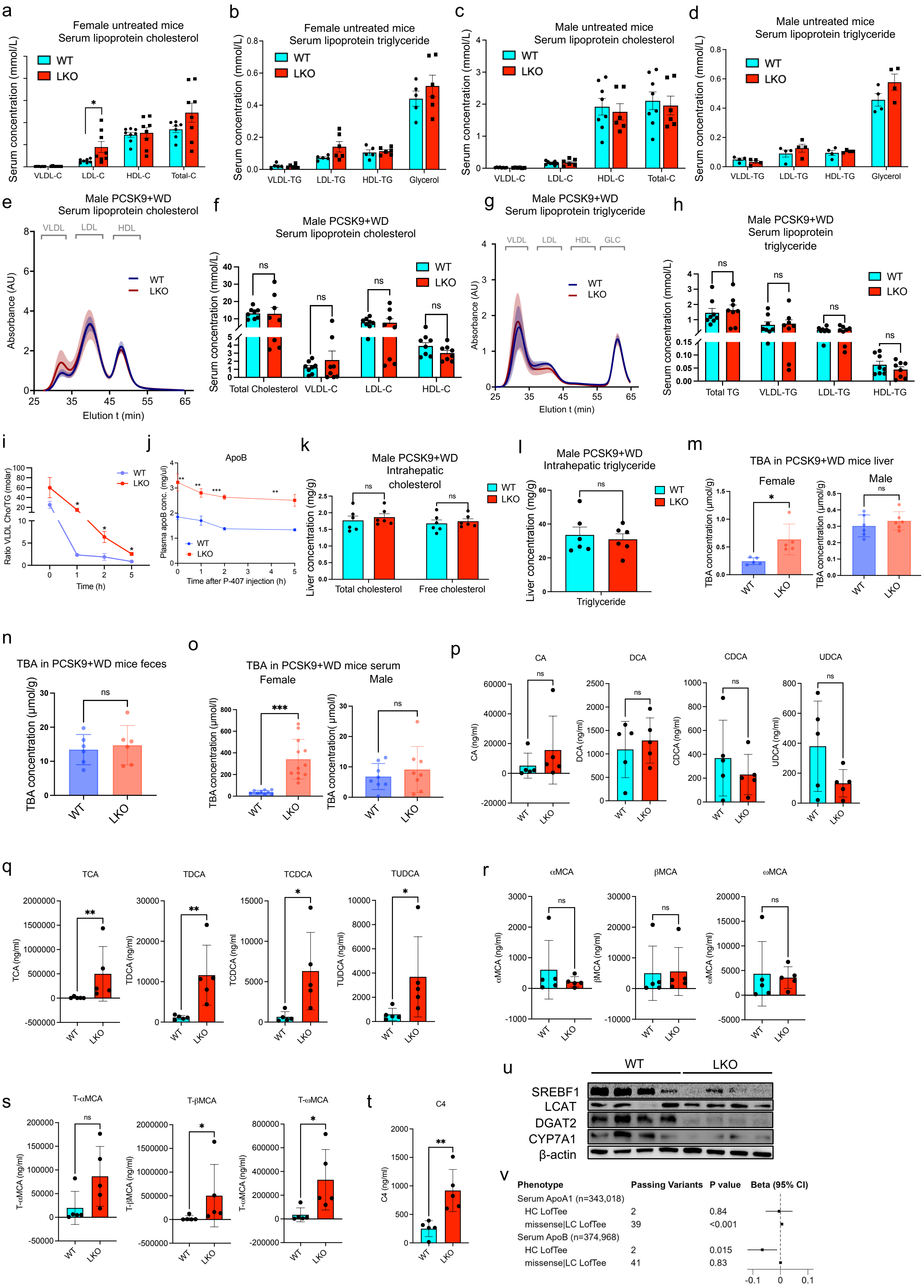
a
Female untreated mice
Serum lipoprotein cholesterol
Serum concentration (mmol/L)
WT
LKO
VLDL-C
LDL-C
HDL-C
Total-C
b
Female untreated mice
Serum lipoprotein triglyceride
Serum concentration (mmol/L)
VLDL-TG
LDL-TG
HDL-TG
Glycerol
c
Male untreated mice
Serum lipoprotein cholesterol
d
Male untreated mice
Serum lipoprotein triglyceride
e
Male PCSK9+WD
Serum lipoprotein cholesterol
VLDL
LDL
HDL
Absorbance (AU)
Elution t (min)
f
Male PCSK9+WD
Serum lipoprotein cholesterol
ns
Total Cholesterol
VLDL-C
LDL-C
HDL-C
g
Male PCSK9+WD
Serum lipoprotein triglyceride
GLC
h
Male PCSK9+WD
Serum lipoprotein
triglyceride
Total TG
VLDL-TG
LDL-TG
HDL-TG
i
Ratio VLDL Cho/TG (molar)
Time (h)
j
ApoB
Plasma apoB conc. (mg/ul)
Time after P-407 injection (h)
k
Male PCSK9+WD
Intrahepatic
cholesterol
Liver concentration (mg/g)
Total cholesterol
Free cholesterol
l
Male PCSK9+WD
Intrahepatic triglyceride
Triglyceride
m
TBA in PCSK9+WD mice liver
Female
Male
TBA concentration (μmol/g)
n
TBA in PCSK9+WD mice feces
TBA concentration (μmol/g)
o
TBA in PCSK9+WD mice serum
Female
Male
TBA concentration (μmol/l)
p
CA
DCA
CDCA
UDCA
CA (ng/ml)
DCA (ng/ml)
CDCA (ng/ml)
UDCA (ng/ml)
q
TCA
TDCA
TCDCA
TUDCA
TCA (ng/ml)
TDCA (ng/ml)
TCDCA (ng/ml)
TUDCA (ng/ml)
r
αMCA
βMCA
ωMCA
αMCA (ng/ml)
βMCA (ng/ml)
ωMCA (ng/ml)
s
T-αMCA
T-βMCA
T-ωMCA
T-αMCA (ng/ml)
T-βMCA (ng/ml)
T-ωMCA (ng/ml)
t
C4
C4 (ng/ml)
u
WT
LKO
SREBF1
LCAT
DGAT2
CYP7A1
β-actin
v
Phenotype
Passing Variants
P value
Beta (95% CI)
Serum ApoA1 (n=343,018)
HC LofTee 2 0.84
missense|LC LofTee 39 <0.001
Serum ApoB (n=374,968)
HC LofTee 2 0.015
missense|LC LofTee 41 0.83
-0.1
0
0.1

Supplementary Figure 2: *Kdm6a* LKO promoted atherosclerosis in vivo. FPLC quantification of **(a)** lipoprotein cholesterol (n=8) and **(b)** triglyceride (n=5 WT, n=6 LKO) in chow diet fed female mice, two-tailed unpaired Student's t test. FPLC quantification of **(c)** lipoprotein cholesterol (n=8 WT, n=6 LKO) and **(d)** triglyceride (n=4 WT and n=4 LKO) analysis in chow diet fed male mice, two-tailed unpaired Student's t test. **(e)** FPLC curve and **(f)** quantification of lipoprotein cholesterol, and **(g)** FPLC curve and **(h)** quantification of lipoprotein triglyceride in male AAV- PCSK9 mice upon (n=8 WT, n=8 LKO), two-tailed unpaired Student's t test. **(i)** Quantification of VLDL cholesterol and triglyceride ratio at different time points after Poloxamer-407 injection in the AAV-PCSK9 mice (n=4 in each group), two-tailed unpaired Student's t test. **(j)** APOB100 ELISA after Poloxamer-407 injection in the AAV-PCSK9 mice (n=5 in each group). Intrahepatic **(k)** cholesterol and **(l)** triglyceride levels in triglyceride in WT and LKO male mice upon AAV- PCSK9 injection and western diet feeding (n=6 in each group), two-tailed unpaired Student's t test. **(m)** Intrahepatic total bile acid levels in AAV-PCSK9 female (n=5 WT, n=5 LKO) and male (n=6 WT, n=6 LKO) mice, two-tailed unpaired Student's t test. **(n)** Feces total bile acid levels in AAV-PCSK9 female WT and LKO (n=6 WT, n=6 LKO) mice, two-tailed unpaired Student's t test. **(o)** Serum total bile acid levels in AAV-PCSK9 female (n=9 WT, n=12 LKO) and male (n=8 WT, n=8 LKO) mice, two-tailed unpaired Student's t test. Serum bile acid composition analysis of **(p) C**A, DCA, CDCA, UDCA, **(q)** T-CA, T-DCA, T-CDCA, T-UDCA, **(r)** αMCA, βMCA, ωMCA, and **(s)** T-αMCA, T-βMCA, T-ωMCA (n=5 in each group), two-tailed unpaired Student's t test. **(t)** Analysis of C4 in the plasma of female WT and LKO mice (n=5 in each group**). (u)** WB analysis of WT (n=4) and LKO (n=4) female AAV-PCSK9 mouse liver biopsies. **(v)** Analysis of patient rare variants association with serum ApoA1 and ApoB in the T2D portal. All data are represented as mean ± s.e.m.**P* < 0.05, ***P* < 0.01, ****P* < 0.001, *****P* < 0.0001.

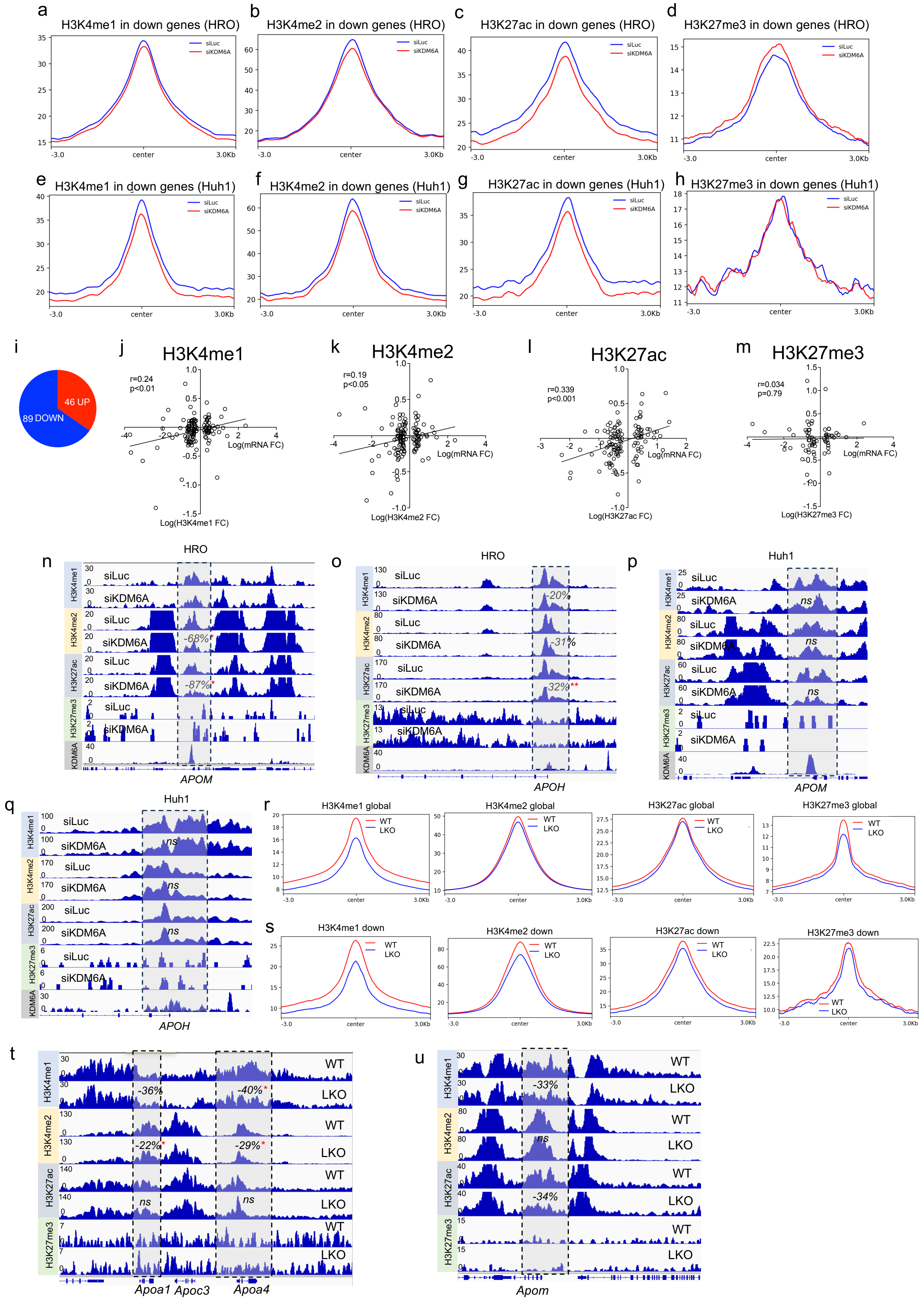
a
H3K4me1 in down genes (HRO)
b
H3K4me2 in down genes (HRO)
c
H3K27ac in down genes (HRO)
d
H3K27me3 in down genes (HRO)
e
H3K4me1 in down genes (Huh1)
f
H3K4me2 in down genes (Huh1)
g
H3K27ac in down genes (Huh1)
h
H3K27me3 in down genes (Huh1)
siLuc
siKDM6A
-3.0
center
3.0Kb
i
46 UP
89 DOWN
j
H3K4me1
r=0.24
p<0.01
Log(mRNA FC)
Log(H3K4me1 FC)
k
H3K4me2
r=0.19
p<0.05
Log(mRNA FC)
Log(H3K4me2 FC)
l
H3K27ac
r=0.339
p<0.001
Log(mRNA FC)
Log(H3K27ac FC)
m
H3K27me3
r=0.034
p=0.79
Log(mRNA FC)
Log(H3K27me3 FC)
n
HRO
-68%*
-87%*
APOM
o
HRO
-20%
-31%
-32%**
APOH
p
Huh1
ns
APOM
q
Huh1
ns
APOH
H3K4me1
H3K4me2
H3K27ac
H3K27me3
KDM6A
r
H3K4me1 global
H3K4me2 global
H3K27ac global
H3K27me3 global
s
H3K4me1 down
H3K4me2 down
H3K27ac down
H3K27me3 down
WT
LKO
t
-36%
-40%*
-22%*
-29%*
ns
Apoa1 Apoc3
Apoa4
u
-33%
ns
-34%
Apom

Supplementary Figure 3: KDM6A regulated lipoprotein and cholesterol metabolic genes are independent of its enzymatic activities. Coverage plot of **(a)** H3K4me1, **(b)** H3K4me2, **(c)** H3K27ac and **(d)** H3K27me3 ChIP-seq in downregulated gene loci upon KDM6A knockdown in HRO cells. Coverage plot of **(e)** H3K4me1, **(f)** H3K4me2, **(g)** H3K27ac and **(h)** H3K27me3 ChIP-seq in downregulated gene loci upon KDM6A knockdown in Huh1 cells. **(i)** Pie chart showing the number and percentage of up- and downregulated LnC genes upon KDM6A knockdown in HRO cells. Correlation of LnC gene expression with **(j)** H3K4me1, **(k)** H3K4me2, **(l)** H3K27ac and **(m)** H3K27me3 changes in HRO cells. Genome browser screenshots of H3K4me1, H3K4me2, H3K27ac and H3K37me3 ChIP-seq results in siLuc and siKDM6A HRO cells at **(n)** *APOM* and **(o)** *APOH* loci in HRO cells. Genome browser screenshots of H3K4me1, H3K4me2, H3K27ac and H3K37me3 ChIP-seq results in siLuc and siKDM6A at **(p)** *APOM* and **(q)** *APOH* loci in Huh1 cells. Coverage plot of H3K4me1, H3K4me2, H3K27ac and H3K27me3 ChIP-seq peaks in the female WT and LKO mouse liver **(r)** genome widely and at **(s)** downregulated gene loci. Genome browser screenshot of H3K4me1, H3K4me2, H3K27ac and H3K27me3 ChIP-seq at **(t)** *Apoa1*/*Apoc3*/*Apoa4* gene cluster and **(u)** *Apom* locus in female WT and LKO mouse liver samples (merged bigwig files from n=3 in each group).

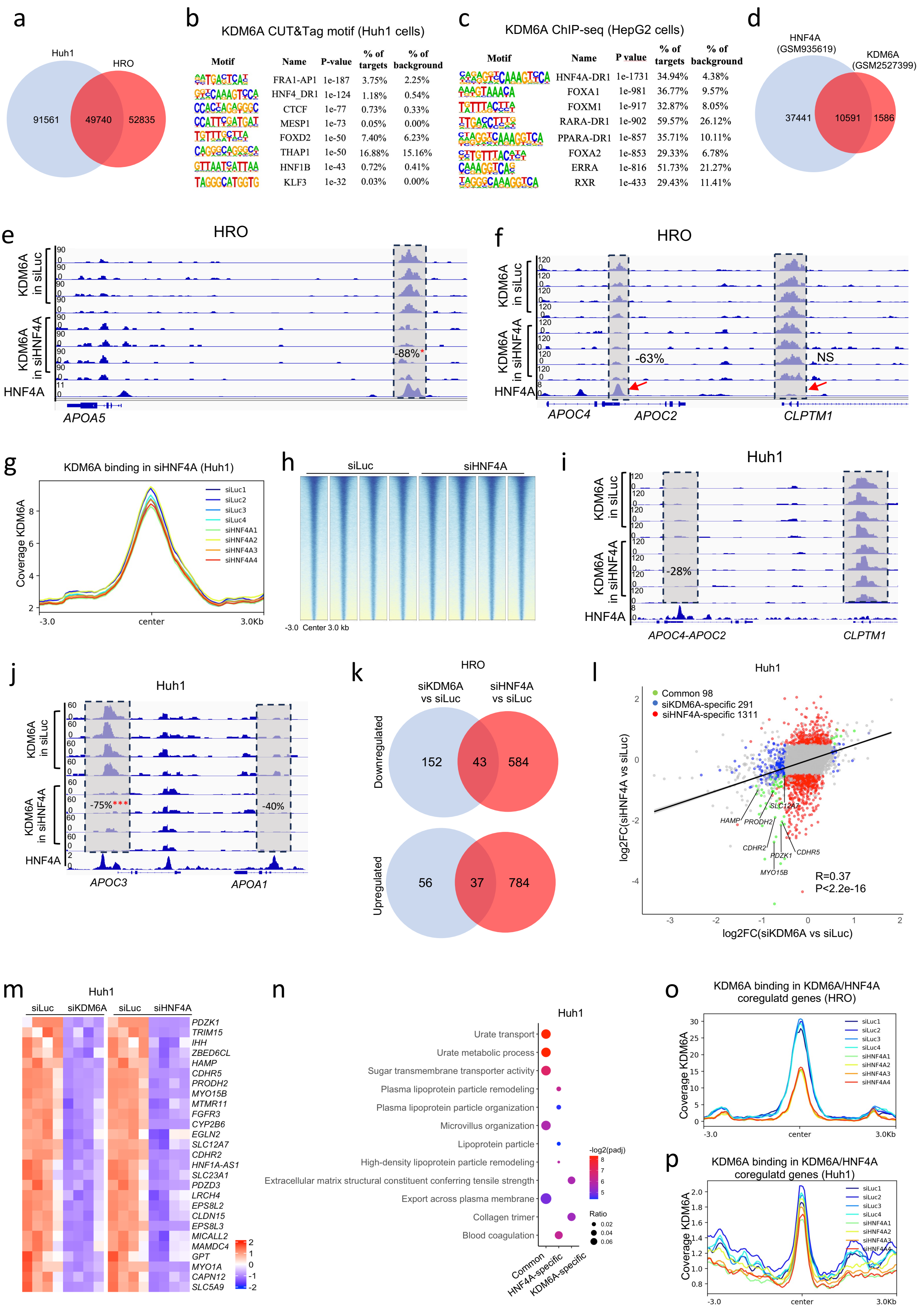
a
Huh1
HRO
91561
49740
52835
b
KDM6A CUT&Tag motif (Huh1 cells)
Motif | Name | P-value | % of targets | % of background
FRA1-AP1 | 1e-187 | 3.75% | 2.25%
HNF4_DR1 | 1e-124 | 1.18% | 0.54%
CTCF | 1e-77 | 0.73% | 0.33%
MESP1 | 1e-73 | 0.05% | 0.00%
FOXD2 | 1e-50 | 7.40% | 6.23%
THAP1 | 1e-50 | 16.88% | 15.16%
HNF1B | 1e-43 | 0.72% | 0.41%
KLF3 | 1e-32 | 0.03% | 0.00%
c
KDM6A ChIP-seq (HepG2 cells)
Motif | Name | P value | % of targets | % of background
HNF4A-DR1 | 1e-1731 | 34.94% | 4.38%
FOXA1 | 1e-981 | 36.77% | 9.57%
FOXM1 | 1e-917 | 32.87% | 8.05%
RARA-DR1 | 1e-902 | 59.57% | 26.12%
PPARA-DR1 | 1e-857 | 35.71% | 10.11%
FOXA2 | 1e-853 | 29.33% | 6.78%
ERRA | 1e-816 | 51.73% | 21.27%
RXR | 1e-433 | 29.43% | 11.41%
d
HNF4A (GSM935619)
KDM6A (GSM2527399)
37441
10591
1586
e
HRO
KDM6A in siLuc
KDM6A in siHNF4A
HNF4A
-88%*
APOA5
f
HRO
KDM6A in siLuc
KDM6A in siHNF4A
HNF4A
-63%
NS
APOC4
APOC2
CLPTM1
g
KDM6A binding in siHNF4A (Huh1)
Coverage KDM6A
siLuc1
siLuc2
siLuc3
siLuc4
siHNF4A1
siHNF4A2
siHNF4A3
siHNF4A4
-3.0
center
3.0Kb
h
siLuc
siHNF4A
-3.0 Center 3.0 kb
i
Huh1
KDM6A in siLuc
KDM6A in siHNF4A
HNF4A
-28%
APOC4-APOC2
CLPTM1
j
Huh1
KDM6A in siLuc
KDM6A in siHNF4A
HNF4A
-75%***
-40%
APOC3
APOA1
k
HRO
siKDM6A vs siLuc
siHNF4A vs siLuc
Downregulated
152
43
584
Upregulated
56
37
784
l
Huh1
Common 98
siKDM6A-specific 291
siHNF4A-specific 1311
log2FC(siHNF4A vs siLuc)
log2FC(siKDM6A vs siLuc)
SLC12A7
HAMP
PRODH2
CDHR2
PDZK1
CDHR5
MYO15B
R=0.37
P<2.2e-16
m
Huh1
siLuc
siKDM6A
siLuc
siHNF4A
PDZK1
TRIM15
IHH
ZBED6CL
HAMP
CDHR5
PRODH2
MYO15B
MTMR11
FGFR3
CYP2B6
EGLN2
SLC12A7
CDHR2
HNF1A-AS1
SLC23A1
PDZD3
LRCH4
EPS8L2
CLDN15
EPS8L3
MICALL2
MAMDC4
GPT
MYO1A
CAPN12
SLC5A9
n
Huh1
Urate transport
Urate metabolic process
Sugar transmembrane transporter activity
Plasma lipoprotein particle remodeling
Plasma lipoprotein particle organization
Microvillus organization
Lipoprotein particle
High-density lipoprotein particle remodeling
Extracellular matrix structural constituent conferring tensile strength
Export across plasma membrane
Collagen trimer
Blood coagulation
Common
HNF4A-specific
KDM6A-specific
-log2(padj)
Ratio
0.02
0.04
0.06
o
KDM6A binding in KDM6A/HNF4A coregulatd genes (HRO)
Coverage KDM6A
-3.0
center
3.0Kb
p
KDM6A binding in KDM6A/HNF4A coregulatd genes (Huh1)
Coverage KDM6A
-3.0
center
3.0Kb

Supplementary Figure 4: KDM6A interplays with HNF4A to control lipoprotein and cholesterol metabolic genes. **(a)** Venn diagram showing the overlapped KDM6A CUT&Tag peaks in Huh1 and HRO cells. Motif analysis of KDM6A **(b)** CUT&Tag peaks in Huh1 and **(c)** ChIP-seq peaks in HepG2 cells. **(d)** Venn diagram showing the overlapped HNF4A and KDM6A ChIP-seq peaks in HepG2 cells. Genome browser screenshot showing KDM6A CUT&Tag peaks at siLuc and siHNF4A in HRO cells, HNF4A ChIP-seq peaks at **(e)** *APOA5* and **(f)** *APOC2*/*APOC4* gene cluster. **(g)** Coverage plot and **(h)** heatmap of the genome wide KDM6A CUT&Tag peak changes upon HNF4A knockdown in Huh1 cells (n=4). Screenshots of genome browser showing KDM6A CUT&Tag peaks in siLuc and siHNF4A transfected Huh1 cells, HNF4A (GSM935619) ChIP-seq peaks at **(i)** *APOC4-APOC2* gene cluster, *CLPTM1* (control gene) locus and **(j)** *APOA1/APOC3* gene cluster. **(k)** Venn diagram to compare the downregulated (upper panel) and upregulated (lower panel) genes in siKDM6A and siHNF4A RNAseq of HRO cells. **(l)** Correlation analysis of RNA-seq between KDM6A (x axis) and HNF4A (y axis) knockdown in Huh1 cells. The blue, red and green dots represent KDM6A- and HNF4A-specific and commonly regulated genes, non-parametric Spearman's test. **(m)** Heatmap showing the representative KDM6A and HNF4A commonly regulated genes in Huh1 cells. **(n)** Gene ontology analysis of KDM6A- and HNF4A-specific and commonly downregulated genes in Huh1 cells. The size of the dots means gene ratio and the color shows -log2(padj). Coverage plot of the KDM6A CUT&Tag peak changes of KDM6A/HNF4A coregulated genes upon HNF4A knockdown in **(o)** HRO and **(p)** Huh1 cells (n=4).

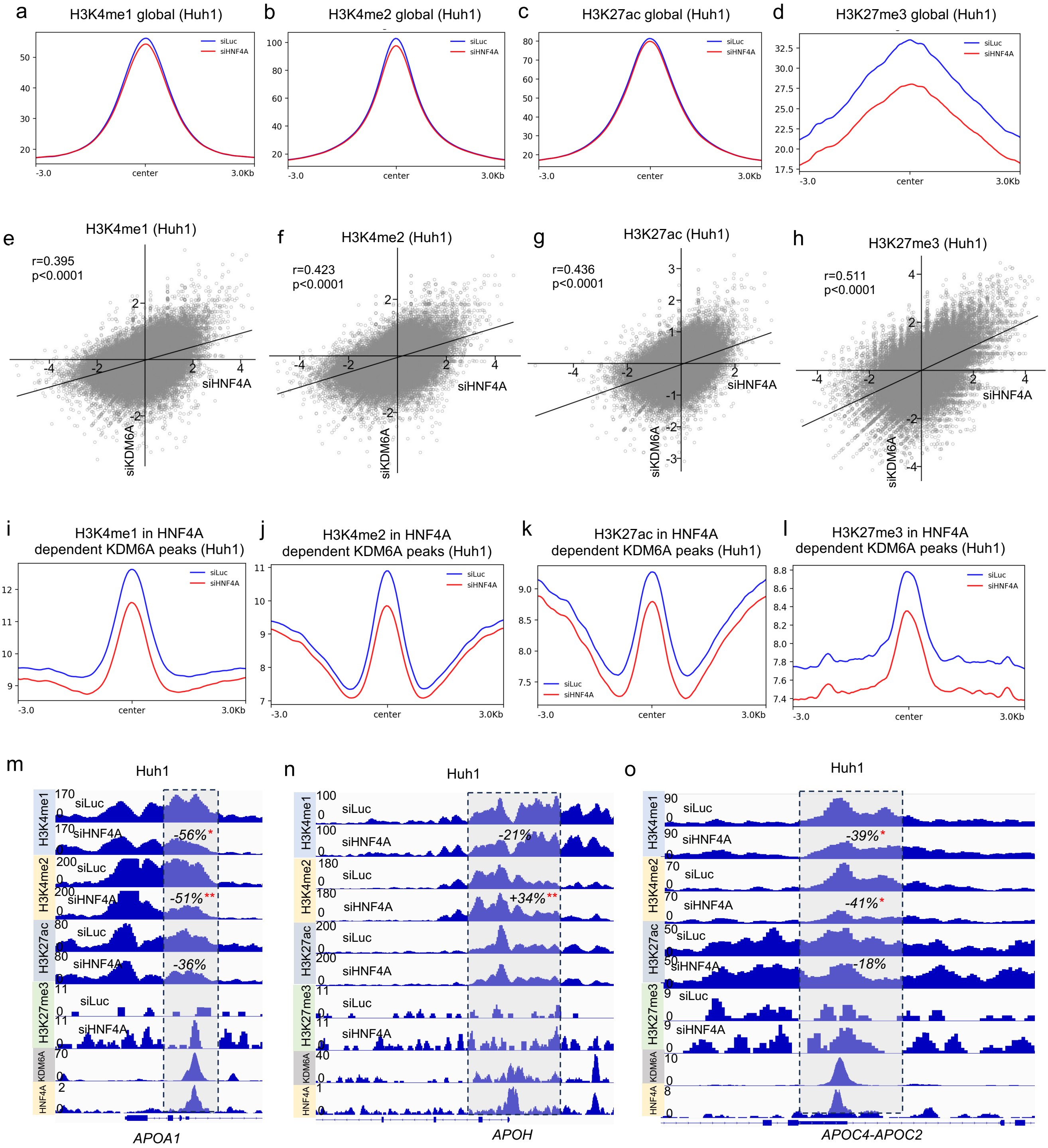
a H3K4me1 global (Huh1)
b H3K4me2 global (Huh1)
c H3K27ac global (Huh1)
d H3K27me3 global (Huh1)
siLuc
siHNF4A
-3.0
center
3.0Kb
e H3K4me1 (Huh1)
r=0.395
p<0.0001
f H3K4me2 (Huh1)
r=0.423
p<0.0001
g H3K27ac (Huh1)
r=0.436
p<0.0001
h H3K27me3 (Huh1)
r=0.511
p<0.0001
siHNF4A
siKDM6A
i H3K4me1 in HNF4A dependent KDM6A peaks (Huh1)
j H3K4me2 in HNF4A dependent KDM6A peaks (Huh1)
k H3K27ac in HNF4A dependent KDM6A peaks (Huh1)
l H3K27me3 in HNF4A dependent KDM6A peaks (Huh1)
m Huh1
n Huh1
o Huh1
H3K4me1
H3K4me2
H3K27ac
H3K27me3
KDM6A
HNF4A
-56%*
-51%**
-36%
-21%
+34%**
-39%*
-41%*
-18%
APOA1
APOH
APOC4-APOC2

Supplementary Figure 5: HNF4A coregulated epigenetic changes with KDM6A in the liver cells. Coverage plots of **(a)** H3K4me1, **(b)** H3K4me2, **(c)** H3K27ac and **(d)** H3K27me3 in siLuc and siHNF4A transfected Huh1 cells. Correlation analysis **(e)** H3K4me1, **(f)** H3K4me2, **(g)** H3K27ac and **(h)** H3K27me3 ChIP-seq changes in siKDM6A and siHNF4A transfected Huh1 cells. x and y axis represents log2(fold change) of each peak. Coverage plots of **(i)** H3K4me1, **(j)** H3K4me2, **(k)** H3K27ac and **(l)** H3K27me3 in HNF4A dependent KDM6A peaks. Genome browser screenshots of H3K4me1, H3K4me2, H3K27ac and H3K27me3 ChIP-seq at **(m)** *APOA1*, **(n)** *APOH* and **(o)** *APOC2/APOC4* gene cluster in siLuc and siHNF4A transfected Huh1 cells (merged bigwig files from n=4 in each group). Spearman's correlation test was performed to compare the siHNF4A and siKDM6A peaks.

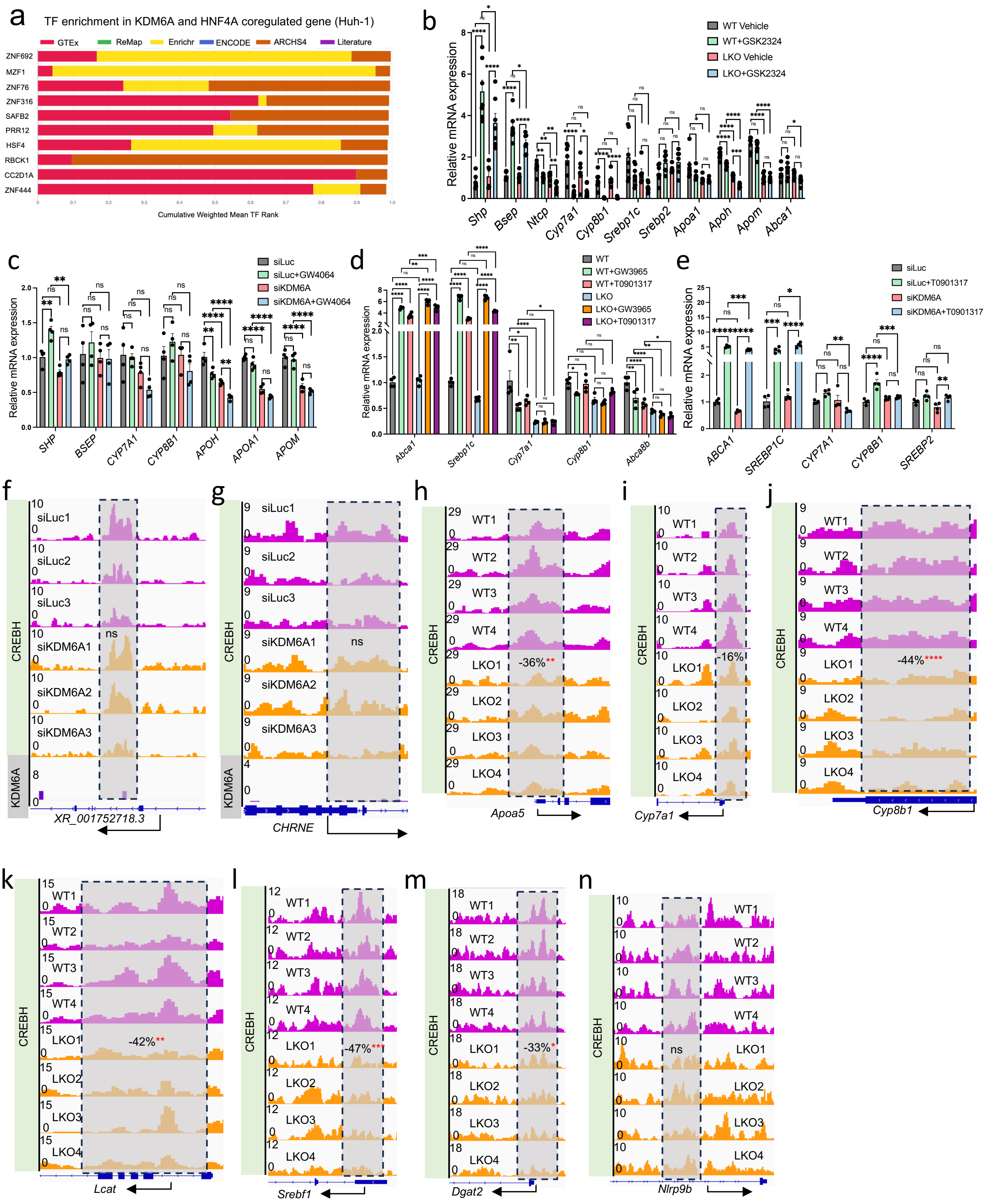
a
TF enrichment in KDM6A and HNF4A coregulated gene (Huh-1)
GTEx
ReMap
Enrichr
ENCODE
ARCHS4
Literature
ZNF692
MZF1
ZNF76
ZNF316
SAFB2
PRR12
HSF4
RBCK1
CC2D1A
ZNF444
Cumulative Weighted Mean TF Rank
b
Relative mRNA expression
WT Vehicle
WT+GSK2324
LKO Vehicle
LKO+GSK2324
Shp
Bsep
Ntcp
Cyp7a1
Cyp8b1
Srebp1c
Srebp2
Apoa1
Apoh
Apom
Abca1
c
Relative mRNA expression
siLuc
siLuc+GW4064
siKDM6A
siKDM6A+GW4064
SHP
BSEP
CYP7A1
CYP8B1
APOH
APOA1
APOM
d
Relative mRNA expression
WT
WT+GW3965
WT+T0901317
LKO
LKO+GW3965
LKO+T0901317
Abca1
Srebp1c
Cyp7a1
Cyp8b1
Abca8b
e
Relative mRNA expression
siLuc
siLuc+T0901317
siKDM6A
siKDM6A+T0901317
ABCA1
SREBP1C
CYP7A1
CYP8B1
SREBP2
f
CREBH
KDM6A
siLuc1
siLuc2
siLuc3
siKDM6A1
siKDM6A2
siKDM6A3
ns
XR_001752718.3
g
CREBH
KDM6A
siLuc1
siLuc2
siLuc3
siKDM6A1
siKDM6A2
siKDM6A3
ns
CHRNE
h
CREBH
WT1
WT2
WT3
WT4
LKO1
LKO2
LKO3
LKO4
-36%**
Apoa5
i
CREBH
-16%
Cyp7a1
j
CREBH
-44%****
Cyp8b1
k
CREBH
-42%**
Lcat
l
CREBH
-47%***
Srebf1
m
CREBH
-33%*
Dgat2
n
CREBH
ns
Nlrp9b

Supplementary Figure 6: KDM6A removal affected CREBH but not FXR or LXR in the liver. **(a)** Transcription factor enrichment analysis of KDM6A and HNF4A coregulated genes in Huh1 cells by CHEA3. qPCR analysis of **(b)** WT and LKO female mouse livers after injection with FXR agonist GSK2324 (n=6 or 7 in each group), one-way ANOVA followed by Tukey's test; **(c)** siLuc and siKDM6A transfected HRO cells treated with FXR agonist GW4064 (n=4 in each group), one-way ANOVA followed by Tukey's test; **(d)** WT and LKO female primary mouse hepatocytes treated with LXR agonists GW3965 and T0901317 (n=4 in each group), one-way ANOVA followed by Tukey's test; **(e)** siLuc and siKDM6A transfected HRO cells treated with LXR agonist T0901317 (n=4 in each group), one-way ANOVA followed by Tukey's test. Genome browser screenshots of CREBH binding in HRO cells at **(f)** *XR_001752718.3* and **(g)** *CHRNE* gene loci (n=3 in each group). CREBH ChIP-seq peaks in AAV-PCSK9 female WT and LKO mice at **(h)** *Apoa5,* **(i)** *Cyp7a1,* **(j)** *Cyp8b1,* **(k)** *Lcat,* **(l)** *Srebf1,* **(m)** *Dgat2,* **(n)** *Nlrp9b* gene cluster (n=4 in each group). All data are represented as mean ± s.e.m. *$P < 0.05$, **$P < 0.01$, ***$P < 0.001$, ****$P < 0.0001$.

Gene	Forward (5'-3')	Reverse (5'-3')
siLuc	CGUACGCGGAAUACUUCGA(dT)(dT)	UCGAAGUAUUCCGCGUACG(dT)(dT)
siKDM6A	GACAACAAGGCAUUACCUU(dT)(dT)	AAGGUAAUGCCUUGUUGUC(dT)(dT)
siHNF4A	GACAACAAGGCAUUACCUU(dT)(dT)	AACACCAUGGAUCUCUUGG(dT)(dT)
siCREBH	CCUGUACGAGUGUUCUCCA(dT)(dT)	UGGAGAACACUCGUACAGG(dT)(dT)
siKMT2C	CAGCUAAGAAGAUCAGUAU(dT)(dT)	AUACUGAUCUUCUUAGCUG(dT)(dT)
siKMT2D	CAUCUACAUGUUCCGAAUA(dT)(dT)	UAUUCGGAACAUGUAGAUG(dT)(dT)
siUTY	GCAUAUCAGAGAUAUUACA(dT)(dT)	UGUAAUAUCUCUGAUAUGC(dT)(dT)

Supplementary Table 1: siRNA for human cell lines.

human primers		
Gene	Forward (5'-3')	Reverse (5'-3')
APOA1	CCCTGGGATCGAGTGAAGGA	CTGGGACACATAGTCTCTGCC
APOM	GCTACCATCCGCATGAAAGAT	CTGGCCTGTCTCATTCAGCA
APOH	ATACAATTACCTGCACGACACAT	GGCCATCCAGAGAATATCCATCA
KDM6A	GGACATGCTGTGTCACATCCT	CTCCTGTTGGTCTCATTTGGTG
HNF4A	CACGGGCAAACACTACGGT	TTGACCTTCGAGTGCTGATCC
APOC2	TGTCCTCCTGGTATTGGGATTT	TGTCTTCTCGTACAGGTTCTGG
UBC	CTGGAAGATGGTCGTACCCTG	GGTCTTGCCAGTGAGTGTCT
HMBS	AGCTTGCTCGCATACAGACG	AGCTCCTTGGTAAACAGGCTT
KMT2C	TGGGTTCACCTAGAGTGTGAC	CTGGCTGTAAACGATCCATCTC
KMT2D	AAAGCCCCCTACCTGCAAAA	TCGGTCAGTCTTACGGGCTA
CREBH	ATGAATACGGATTTAGCTGCTGG	AGGAAGTCGTCAGAGTCGGG
UTY	CGCAGTGTCGCTCACTACC	GTCAGGCTAACAGACTCCTCTT
SHP	CTCTTCAACCCCGATGTGCCA	CAGGGTTCCAGGACTTCACA
BSEP	CATGTCACTCCCAGAGAAATATGA	CAATGCGTTGTTTCTCCCCG
CYP7A1	GAGAAGGCAAACGGGTGAAC	GGATTGGCACCAAATTGCAGA
CYP8B1	GAAGCGCATGAGGACCAAG	TTGCATATTGCCCAAAGTCTAGT
ABCA1	ACCCACCCTATGAACAACATGA	GAGTCGGGTAACGGAAACAGG
SREBP1C	CGGAGCCATGGATTGCACTTTC	GATGCTCAGTGGCACTGACTCTTC
SREBP2	AACGGTCATTCACCCAGGTC	GGCTGAAGAATAGGAGTTGCC
mice primers		
Apoa1	GCTCAAGAGCAACCCTACCTT	GCTTTCTCGCCAAGTGTCTTC
Apom	TAACTCCATGAATCAGTGCCCT	CCCGCAATAAAGTACCACAGG
Apoh	TGCCATGTTGCTATTGCAGGA	GGCTTGCAGGAGTAGACAATCT
Kdm6a	CGGGCGGACAAAAGAAGAAC	CATAGACTTGCATCAGATCCTCC
Shp	CTCTTCAACCCAGATGTGCCA	CAGGGCTCCAAGACTTCACA
Bsep	CGCATTGCTATTGCTCGGG	CAAGCTGCACTGTCTTTTCACTT
Ntcp	TTGCGCCATAGGGATCTTCC	ATCATGCCTGCCTTGAGGAC
Srebp1c	ATCGGCGCGGAAGCTGTCGGGGTAGCGTC	ACTGTCTTGGTTGTTGATGAGCTGGAGCAT
Srebp2	GCAGCAACGGGACCATTCT	CCCCATGACTAAGTCCTTCAACT
Abca1	GCTTGTTGGCCTCAGTTAAGG	GTAGCTCAGGCGTACAGAGAT
Baat	CCAGTCTTCTGGCAAGTCGT	GCCGAGGACCTTAGGATGTC
Pcsk9	TTGCCCCATGTGGAGTACATT	GGGAGCGGTCTTCCTCTGT
Ldlr	TGACTCAGACGAACAAGGCTG	ATCTAGGCAATCTCGGTCTCC
Cyp7a1	GCTAAGACGCACCTCGTGAT	TCAGGGCTCCTGATCATTTGAA
Cyp8b1	TCTTTGCCCTCAGCGAGATG	CTAGGCTGTGAGGTGCCAAA
Rplpo	AGATTCGGGATATGCTGTTGGC	TCGGGTCCTAGACCAGTGTTC
Dgat2	TTCCTGGCATAAGGCCCTATT	CCTCCAGACATCAGGTACTCG
Abcc6	TGCGGCCTATCACTTGCTC	CCAGCACCATTTTGGTTTTGAA
Apoa5	TCCTCGCAGTGTTCGCAAG	CGAAGCTGCCTTTCAGGTTCT
Scap	CCGAGCATTCCAACTGGTG	CCATGTTCGGGAAGTAGGCT
Lcat	GTAACCACACACGGCCTGTC	TCTTACGGTAGCACATCCAGTT
Mrp2	ACATCTGCTTCCCTTGAGGC	AGAGATTCCAAAAAGTAGAGTTGC

Supplementary table 2: qPCR primers for human and mice.
