# Supplementary material for: Sex-specific KDM6A-HNF4A-CREBH network controls lipoprotein cholesterol metabolism and atherosclerosis via epigenetic reprograming of hepatocytes
Source: Nat Commun. 2026 Mar 23;17:3945. doi: 10.1038/s41467-026-70846-w (PMC13133126; doi:10.1038/s41467-026-70846-w)
Supplement: Supplementary file 2 — Reporting summary [file 41467_2026_70846_MOESM2_ESM.pdf]

nature portfolio

Corresponding author(s): *Double-anonymous peer review submissions: write DAPR and your manuscript number here instead of author names.*

Last updated by author(s): Feb 26, 2026

Reporting Summary

Nature Portfolio wishes to improve the reproducibility of the work that we publish. This form provides structure for consistency and transparency in reporting. For further information on Nature Portfolio policies, see our Editorial Policies and the Editorial Policy Checklist.

Statistics

For all statistical analyses, confirm that the following items are present in the figure legend, table legend, main text, or Methods section.

n/a	Confirmed	
☐	☒	The exact sample size (*n*) for each experimental group/condition, given as a discrete number and unit of measurement
☐	☒	A statement on whether measurements were taken from distinct samples or whether the same sample was measured repeatedly
☐	☒	The statistical test(s) used AND whether they are one- or two-sided<br>*Only common tests should be described solely by name; describe more complex techniques in the Methods section.*
☐	☒	A description of all covariates tested
☐	☒	A description of any assumptions or corrections, such as tests of normality and adjustment for multiple comparisons
☐	☒	A full description of the statistical parameters including central tendency (e.g. means) or other basic estimates (e.g. regression coefficient) AND variation (e.g. standard deviation) or associated estimates of uncertainty (e.g. confidence intervals)
☐	☒	For null hypothesis testing, the test statistic (e.g. F, t, r) with confidence intervals, effect sizes, degrees of freedom and P value noted<br>*Give P values as exact values whenever suitable.*
☒	☐	For Bayesian analysis, information on the choice of priors and Markov chain Monte Carlo settings
☒	☐	For hierarchical and complex designs, identification of the appropriate level for tests and full reporting of outcomes
☒	☐	Estimates of effect sizes (e.g. Cohen's *d*, Pearson's *r*), indicating how they were calculated

Our web collection on statistics for biologists contains articles on many of the points above.

Software and code

Policy information about availability of computer code

Data collection

ChIP-seq data collection. Fresh liver tissues (chopped into small pieces) and HRO or Huh1 cells were crosslinked with 1% formaldehyde (28906, ThermoFisher) in PBS for 10 minutes for histone modifications. For TFs or coregulators, tissues/cells were double crosslinked with 2 mM disuccinimidyl glutarate (DSG) (20593, ThermoFisher) for 30 minutes, followed by 1% formaldehyde for 10 minutes. The reaction was stopped with glycine at a final concentration of 0.125 M for 5 minutes. Liver pieces were disaggregated in ice-cold PBS with protease inhibitor (12352204, Roche) using a dounce homogenizer, first with a loose pestle and then with a tight pestle (FB56691, Fisher Science). Nuclei were isolated using three lysis buffers: lysis buffer 1 (50 mM Hepes–KOH, pH 7.5, 140 mM NaCl, 1 mM EDTA, 10% glycerol, 0.5% IGEPAL CA-630, and 0.25% Triton X-100), lysis buffer 2 (10 mM Tris–HCl, pH 8.0, 200 mM NaCl, 1 mM EDTA, and 0.5 mM EGTA), and lysis buffer 3 (10 mM Tris–HCl, pH 8.0, 100 mM NaCl, 1 mM EDTA, 0.5 mM EGTA, 0.1% Na-deoxycholate, and 0.5% N-Lauroylsarcosine). The samples were then sonicated for 30 minutes (30 seconds ON/30 seconds OFF) using the Bioruptor Pico (Diagenode). Protein A Dynabeads (10001D, Invitrogen) were incubated overnight with the antibodies. Each lysate was immunoprecipitated with the following antibodies: control rabbit IgG (sc-2027, Santa Cruz, 1–5 µg), anti-H3K27ac (ab177178, Abcam, 1 µg), anti-H3K4me1 (ab8895, Abcam, 1 µg), and anti-H3K4me2 (ab7766, Abcam, 1 µg), anti-CREBH (HPA040671, Sigma Aldrich, 1µg). The immunoprecipitated Protein A beads were washed 3 times with lysis buffer and sent for proteomics analysis (RIME37). Formaldehyde cross-linking was reversed overnight at 65°C, and the immunoprecipitated DNA was purified using the QIAquick PCR purification kit (Qiagen).
For ChIPseq library preparation, the same ChIP protocol was followed, but the ChIP DNA was purified using the ChIP DNA Clean and Concentrator Capped Zymo-Spin I (Zymo Research) purification kit. Two to four ChIPs were pooled during the final purification step to obtain concentrated material. For library preparation and sequencing, 2–10 ng of ChIPed DNA was processed using the Rubicon ThruPLEX DNA-seq kit (TAKARA) following standard protocols, and sequenced on the NovaSeq X Plus Series (150PE reads, Novogene).
CUT&Tag sample preparation. CUT&Tag sample preparation was performed following published protocols from the Henikoff lab. Approximately 500,000 HRO or Huh1 cells were harvested and counted and were resuspended in Wash Buffer and mixed with Concanavalin A-coated magnetic beads. The mixture was incubated at room temperature to allow binding. Bead-bound cells were then washed and

incubated with the primary antibodies specific to the target protein, followed by a secondary antibody (PA5-31828, EpiCypher) for bridging. Next, the bead-bound cells were incubated with pAG-Tn5 (79561, EpiCypher) in the presence of digitonin for tethering. After a brief incubation to allow the transposome to tether to chromatin sites targeted by antibodies, cells were washed and subjected to magnesium activation to induce tagmentation. This step facilitated DNA cleavage and simultaneous tagging with sequencing adapters. Following tagmentation, DNA was extracted and quantified using Qubit fluorometer (Thermo Fisher) following the instructions. Libraries were PCR-amplified with barcoded primers, cleaned up using SPRI beads, and subjected to quality control checks, including size selection and quantification. The prepared libraries were sequenced on the NovaSeq X Plus Series (150PE reads, Novogene, United Kingdom).
RNA-seq data collection. RNA was extracted from mouse liver biopsies or human cell lines using the kit stated above. RNA quality was assessed using the 5400 Fragment Analyzer System (Agilent). mRNA library preparation was carried out using the Novogene NGS RNA Library Prep Set (PT042, Novovene) following the manufacturer's protocol. RNA-seq libraries were sequenced on the NovaSeq X Plus Series (PE150) platform at NOVOGENE (Cambridge, United Kingdom).

Data analysis

ChIP-seq data analysis. Computations of mice data were performed using resources provided by Galaxy. The computations of human data was enabled by resources in project sens2024555 provided by the National Academic Infrastructure for Supercomputing in Sweden (NAISS) at UPPMAX. Analysis was conducted as previously described. Sequencing files (fastq) provided by Novogene (Cambridge, United Kingdom), along with the raw data from published ChIP-seq datasets (KDM6A: GSE95890; HNF4A: GSE31477), were aligned to the NCBI38/mm10 version of the mouse reference genome or GRCh38/hg38 using Bowtie2. Peaks were identified using the HOMER package.
Peak heights were normalized to the total number of uniquely mapped reads and displayed in the Integrative Genomics Viewer (IGV) as the number of tags per 10 million tags. For statistical analysis of the peaks, raw tag counts were imported into R and Bioconductor, and the edgeR package was used to identify potential differential-binding sites.
CUT&Tag data analysis. Sequencing data was processed using standard pipelines. Sequencing files (fastq) provided by Novogene (Cambridge, United Kingdom) were aligned to the GRCh38/hg38 version of the human reference genome using Bowtie2. Duplicate reads were removed, and peaks were called using the HOMER package to identify regions of significant enrichment. SEACR is used to call peaks and enriched regions from chromatin profiling data. ChIPseqSpikeInFree is used to normalize Data.
RNA-seq data analysis: Preprocessed reads were aligned to the NCBI38/mm10 (for mouse) or GRCh38/hg38 (for human) genomes using the HISAT2 program, and read counts were determined using featureCounts v1.5.0-p3. Raw tag counts were imported into R and Bioconductor, and differential gene expression was analyzed using the edgeR package.

For manuscripts utilizing custom algorithms or software that are central to the research but not yet described in published literature, software must be made available to editors and reviewers. We strongly encourage code deposition in a community repository (e.g. GitHub). See the Nature Portfolio guidelines for submitting code & software for further information.

Data

Policy information about availability of data

All manuscripts must include a data availability statement. This statement should provide the following information, where applicable:

- Accession codes, unique identifiers, or web links for publicly available datasets
- A description of any restrictions on data availability
- For clinical datasets or third party data, please ensure that the statement adheres to our policy

Gene expression RNA-seq data, CUT&Tag and ChIP-seq data have been deposited at the NCBI Gene Expression Omnibus (GEO) accession numbers are GSE287680, GSE287688 and GSE287736. Other data are available from the corresponding author upon request.

Research involving human participants, their data, or biological material

Policy information about studies with human participants or human data. See also policy information about sex, gender (identity/presentation), and sexual orientation and race, ethnicity and racism.

Reporting on sex and gender

Use the terms sex (biological attribute) and gender (shaped by social and cultural circumstances) carefully in order to avoid confusing both terms. Indicate if findings apply to only one sex or gender; describe whether sex and gender were considered in study design; whether sex and/or gender was determined based on self-reporting or assigned and methods used.
Provide in the source data disaggregated sex and gender data, where this information has been collected, and if consent has been obtained for sharing of individual-level data; provide overall numbers in this Reporting Summary. Please state if this information has not been collected.
Report sex- and gender-based analyses where performed, justify reasons for lack of sex- and gender-based analysis.

Reporting on race, ethnicity, or other socially relevant groupings

Please specify the socially constructed or socially relevant categorization variable(s) used in your manuscript and explain why they were used. Please note that such variables should not be used as proxies for other socially constructed/relevant variables (for example, race or ethnicity should not be used as a proxy for socioeconomic status).
Provide clear definitions of the relevant terms used, how they were provided (by the participants/respondents, the researchers, or third parties), and the method(s) used to classify people into the different categories (e.g. self-report, census or administrative data, social media data, etc.)
Please provide details about how you controlled for confounding variables in your analyses.

Population characteristics

Describe the covariate-relevant population characteristics of the human research participants (e.g. age, genotypic information, past and current diagnosis and treatment categories). If you filled out the behavioural & social sciences study design questions and have nothing to add here, write "See above."

Recruitment

Describe how participants were recruited. Outline any potential self-selection bias or other biases that may be present and how these are likely to impact results.

Ethics oversight | Identify the organization(s) that approved the study protocol.

Note that full information on the approval of the study protocol must also be provided in the manuscript.

Field-specific reporting

Please select the one below that is the best fit for your research. If you are not sure, read the appropriate sections before making your selection.

☒ Life sciences ☐ Behavioural & social sciences ☐ Ecological, evolutionary & environmental sciences

For a reference copy of the document with all sections, see nature.com/documents/nr-reporting-summary-flat.pdf

Life sciences study design

All studies must disclose on these points even when the disclosure is negative.

Sample size	Sample size was not pre-determined. For most of the mice experiments, the sample size is at least 5 in each group. For ChIP-seq and cell experiments, at least biological triplicates were used in repeated experiments
Data exclusions	Data exclusion is determined by testing the significant outlier using Grubbs' test.
Replication	All the data in the manuscript is at least performed in replicate and repeated at least twice.
Randomization	The animals were selected to ensure 1)they were from the same breedings; 2) they were of same age; and 3) they had similar body weight; and they were then randomly allocated to each experimental group.
Blinding	The experiments were performed by technicians and half blind to the researcher.

Reporting for specific materials, systems and methods

We require information from authors about some types of materials, experimental systems and methods used in many studies. Here, indicate whether each material, system or method listed is relevant to your study. If you are not sure if a list item applies to your research, read the appropriate section before selecting a response.

Materials & experimental systems

n/a	Involved in the study	
☐	☒	Antibodies
☐	☒	Eukaryotic cell lines
☐	☐	Palaeontology and archaeology
☐	☒	Animals and other organisms
☐	☐	Clinical data
☐	☐	Dual use research of concern
☐	☐	Plants

Methods

n/a	Involved in the study	
☐	☒	ChIP-seq
☐	☐	Flow cytometry
☐	☐	MRI-based neuroimaging

Antibodies

Antibodies used	control rabbit IgG (sc-2027, Santa Cruz), anti-H3K27ac (ab177178, Abcam), anti-H3K4me1 (ab8895, Abcam), anti-H3K4me2 (ab7766, Abcam), anti-H3K27me3 (07-449,Millipore), anti-CREBH (HPA040671, Sigma Aldrich), anti-KDM6A (PA5-31828, invitrogen), anti-Srebf1 (14088-1-AP, Proteintech), anti-Lcat (12243-1-AP, Proteintech), anti-Dgat2 (17100-1-AP, Proteintech), anti-Cyp7a1 (ab65596, Abcam) and anti-Beta-actin (ab8226, Abcam).
Validation	control rabbit IgG (sc-2027, Santa Cruz), anti-H3K27ac (ab177178, Abcam), anti-H3K4me1 (ab8895, Abcam), anti-H3K4me2 (ab7766, Abcam), anti-H3K27me3 (07-449,Millipore) and anti-CREBH (HPA040671, Sigma Aldrich) were validated in ChIP-seq using human cell lines and mice liver tissues. anti-KDM6A (PA5-31828, invitrogen) antibodies were validated in CUT&Tag using human cell lines. anti-Srebf1 (14088-1-AP, Proteintech), anti-Lcat (12243-1-AP, Proteintech), anti-Dgat2 (17100-1-AP, Proteintech), anti-Cyp7a1 (ab65596, Abcam) and anti-Beta-actin (ab8226, Abcam) antibodies were validated in Western blotting using mice liver tissues.

Eukaryotic cell lines

Policy information about cell lines and Sex and Gender in Research

Cell line source(s)	Huh7 (RRID: CVCL_0336, Cytion-300156), SNU-182 (RRID: CVCL_0090, Cytion-305119), Huh-1 (RRID: CVCL_2956, JCRB-JCRB0199, established by Hoh,H.) and HROHep03 (RRID: CVCL_2U72, Cytion-300197), Primary Human Aortic Smooth Muscle Cells (ATCC, PCS-100-012)

Authentication	*Describe the authentication procedures for each cell line used OR declare that none of the cell lines used were authenticated.*
Mycoplasma contamination	All the cells have been tested for mycoplasma contaminations.
Commonly misidentified lines (See ICLAC register)	*Name any commonly misidentified cell lines used in the study and provide a rationale for their use.*

Palaeontology and Archaeology

Specimen provenance	*Provide provenance information for specimens and describe permits that were obtained for the work (including the name of the issuing authority, the date of issue, and any identifying information). Permits should encompass collection and, where applicable, export.*
Specimen deposition	*Indicate where the specimens have been deposited to permit free access by other researchers.*
Dating methods	*If new dates are provided, describe how they were obtained (e.g. collection, storage, sample pretreatment and measurement), where they were obtained (i.e. lab name), the calibration program and the protocol for quality assurance OR state that no new dates are provided.*

☐ Tick this box to confirm that the raw and calibrated dates are available in the paper or in Supplementary Information.

Ethics oversight	*Identify the organization(s) that approved or provided guidance on the study protocol, OR state that no ethical approval or guidance was required and explain why not.*

Note that full information on the approval of the study protocol must also be provided in the manuscript.

Animals and other research organisms

Policy information about studies involving animals; ARRIVE guidelines recommended for reporting animal research, and Sex and Gender in Research

Laboratory animals	Kdm6aflox/flox mice were developed in Cyagen using a targeting construct which contains loxp sites flanking exon 5 of Kdm6a. To create the LKO mice, the Kdm6aflox/flox mice were crossed with Alb-Cre mice (B6.Cg-Speer6-ps1Tg(Alb-cre)21Mgn/J) obtained from Jackson Laboratory (stock no. 003574). Both Kdm6a flox/flox and the Alb-Cre mice were bred with wild type C57BJ6 mice for at least 9 generations before breeding. The paired Kdm6aflox/floxAlb-Cre-/- mice were used as negative controls.
Wild animals	*Provide details on animals observed in or captured in the field; report species and age where possible. Describe how animals were caught and transported and what happened to captive animals after the study (if killed, explain why and describe method; if released, say where and when) OR state that the study did not involve wild animals.*
Reporting on sex	All animal experiments were conducted by separating the animals by gender.
Field-collected samples	*For laboratory work with field-collected samples, describe all relevant parameters such as housing, maintenance, temperature, photoperiod and end-of-experiment protocol OR state that the study did not involve samples collected from the field.*
Ethics oversight	All animal experiments were approved by the respective national ethical boards (Swedish Board of Agriculture, Stockholm South, 05517-2022) and conducted in accordance with the guidelines stated in the International Guiding Principles for Biomedical Research Involving Animals, developed by the Council for International Organizations of Medical Sciences (CIOMS). All mice strains were bred and maintained at the Center for Comparative Medicine at Karolinska Institutet and University Hospital (PKL, Huddinge, Sweden)

Note that full information on the approval of the study protocol must also be provided in the manuscript.

Clinical data

Policy information about clinical studies

All manuscripts should comply with the ICMJE guidelines for publication of clinical research and a completed CONSORT checklist must be included with all submissions.

Clinical trial registration	*Provide the trial registration number from ClinicalTrials.gov or an equivalent agency.*
Study protocol	*Note where the full trial protocol can be accessed OR if not available, explain why.*
Data collection	*Describe the settings and locales of data collection, noting the time periods of recruitment and data collection.*
Outcomes	*Describe how you pre-defined primary and secondary outcome measures and how you assessed these measures.*

Dual use research of concern

Policy information about dual use research of concern

Hazards

Could the accidental, deliberate or reckless misuse of agents or technologies generated in the work, or the application of information presented in the manuscript, pose a threat to:

No	Yes	
☒	☐	Public health
☒	☐	National security
☒	☐	Crops and/or livestock
☒	☐	Ecosystems
☒	☐	Any other significant area

Experiments of concern

Does the work involve any of these experiments of concern:

No	Yes	
☒	☐	Demonstrate how to render a vaccine ineffective
☒	☐	Confer resistance to therapeutically useful antibiotics or antiviral agents
☒	☐	Enhance the virulence of a pathogen or render a nonpathogen virulent
☒	☐	Increase transmissibility of a pathogen
☒	☐	Alter the host range of a pathogen
☒	☐	Enable evasion of diagnostic/detection modalities
☒	☐	Enable the weaponization of a biological agent or toxin
☒	☐	Any other potentially harmful combination of experiments and agents

Plants

Seed stocks

Report on the source of all seed stocks or other plant material used. If applicable, state the seed stock centre and catalogue number. If plant specimens were collected from the field, describe the collection location, date and sampling procedures.

Novel plant genotypes

Describe the methods by which all novel plant genotypes were produced. This includes those generated by transgenic approaches, gene editing, chemical/radiation-based mutagenesis and hybridization. For transgenic lines, describe the transformation method, the number of independent lines analyzed and the generation upon which experiments were performed. For gene-edited lines, describe the editor used, the endogenous sequence targeted for editing, the targeting guide RNA sequence (if applicable) and how the editor was applied.

Authentication

Describe any authentication procedures for each seed stock used or novel genotype generated. Describe any experiments used to assess the effect of a mutation and, where applicable, how potential secondary effects (e.g. second site T-DNA insertions, mosiacism, off-target gene editing) were examined.

ChIP-seq

Data deposition

☒ Confirm that both raw and final processed data have been deposited in a public database such as GEO.

☒ Confirm that you have deposited or provided access to graph files (e.g. BED files) for the called peaks.

Data access links
May remain private before publication.

https://www.ncbi.nlm.nih.gov/geo/query/acc.cgi?acc=GSE287736. Secure token of ChIP-seq Data(GSE287736) for reviewers: qpkpaceolxithix

Files in database submission

NC1_K4me1_1.fq.gz NC1_K4me1_2.fq.gz NC1_K4me1.bigwig
NC2_K4me1_1.fq.gz NC2_K4me1_2.fq.gz NC2_K4me1.bigwig
NC3_K4me1_1.fq.gz NC3_K4me1_2.fq.gz NC3_K4me1.bigwig
NC4_K4me1_1.fq.gz NC4_K4me1_2.fq.gz NC4_K4me1.bigwig
KA1_K4me1_1.fq.gz KA1_K4me1_2.fq.gz KA1_K4me1.bigwig
KA2_K4me1_1.fq.gz KA2_K4me1_2.fq.gz KA2_K4me1.bigwig
KA3_K4me1_1.fq.gz KA3_K4me1_2.fq.gz KA3_K4me1.bigwig
KA4_K4me1_1.fq.gz KA4_K4me1_2.fq.gz KA4_K4me1.bigwig

HA1_K4me1_1.fq.gz HA1_K4me1_2.fq.gz HA1_K4me1.bigwig
HA2_K4me1_1.fq.gz HA2_K4me1_2.fq.gz HA2_K4me1.bigwig
HA3_K4me1_1.fq.gz HA3_K4me1_2.fq.gz HA3_K4me1.bigwig
HA4_K4me1_1.fq.gz HA4_K4me1_2.fq.gz HA4_K4me1.bigwig
NC1_K4me2_1.fq.gz NC1_K4me2_2.fq.gz NC1_K4me2.bigwig
NC2_K4me2_1.fq.gz NC2_K4me2_2.fq.gz NC2_K4me2.bigwig
NC3_K4me2_1.fq.gz NC3_K4me2_2.fq.gz NC3_K4me2.bigwig
NC4_K4me2_1.fq.gz NC4_K4me2_2.fq.gz NC4_K4me2.bigwig
KA1_K4me2_1.fq.gz KA1_K4me2_2.fq.gz KA1_K4me2.bigwig
KA2_K4me2_1.fq.gz KA2_K4me2_2.fq.gz KA2_K4me2.bigwig
KA3_K4me2_1.fq.gz KA3_K4me2_2.fq.gz KA3_K4me2.bigwig
KA4_K4me2_1.fq.gz KA4_K4me2_2.fq.gz KA4_K4me2.bigwig
HA1_K4me2_1.fq.gz HA1_K4me2_2.fq.gz HA1_K4me2.bigwig
HA2_K4me2_1.fq.gz HA2_K4me2_2.fq.gz HA2_K4me2.bigwig
HA3_K4me2_1.fq.gz HA3_K4me2_2.fq.gz HA3_K4me2.bigwig
HA4_K4me2_1.fq.gz HA4_K4me2_2.fq.gz HA4_K4me2.bigwig
NC1_K27ac_1.fq.gz NC1_K27ac_2.fq.gz NC1_K27ac.bigwig
NC2_K27ac_1.fq.gz NC2_K27ac_2.fq.gz NC2_K27ac.bigwig
NC3_K27ac_1.fq.gz NC3_K27ac_2.fq.gz NC3_K27ac.bigwig
NC4_K27ac_1.fq.gz NC4_K27ac_2.fq.gz NC4_K27ac.bigwig
KA1_K27ac_1.fq.gz KA1_K27ac_2.fq.gz KA1_K27ac.bigwig
KA2_K27ac_1.fq.gz KA2_K27ac_2.fq.gz KA2_K27ac.bigwig
KA3_K27ac_1.fq.gz KA3_K27ac_2.fq.gz KA3_K27ac.bigwig
KA4_K27ac_1.fq.gz KA4_K27ac_2.fq.gz KA4_K27ac.bigwig
HA1_K27ac_1.fq.gz HA1_K27ac_2.fq.gz HA1_K27ac.bigwig
HA2_K27ac_1.fq.gz HA2_K27ac_2.fq.gz HA2_K27ac.bigwig
HA3_K27ac_1.fq.gz HA3_K27ac_2.fq.gz HA3_K27ac.bigwig
HA4_K27ac_1.fq.gz HA4_K27ac_2.fq.gz HA4_K27ac.bigwig
NC1_K27me3_1.fq.gz NC1_K27me3_2.fq.gz NC1_K27me3.bigwig
NC2_K27me3_1.fq.gz NC2_K27me3_2.fq.gz NC2_K27me3.bigwig
NC3_K27me3_1.fq.gz NC3_K27me3_2.fq.gz NC3_K27me3.bigwig
NC4_K27me3_1.fq.gz NC4_K27me3_2.fq.gz NC4_K27me3.bigwig
KA1_K27me3_1.fq.gz KA1_K27me3_2.fq.gz KA1_K27me3.bigwig
KA2_K27me3_1.fq.gz KA2_K27me3_2.fq.gz KA2_K27me3.bigwig
KA3_K27me3_1.fq.gz KA3_K27me3_2.fq.gz KA3_K27me3.bigwig
KA4_K27me3_1.fq.gz KA4_K27me3_2.fq.gz KA4_K27me3.bigwig
HA1_K27me3_1.fq.gz HA1_K27me3_2.fq.gz HA1_K27me3.bigwig
HA2_K27me3_1.fq.gz HA2_K27me3_2.fq.gz HA2_K27me3.bigwig
HA3_K27me3_1.fq.gz HA3_K27me3_2.fq.gz HA3_K27me3.bigwig
HA4_K27me3_1.fq.gz HA4_K27me3_2.fq.gz HA4_K27me3.bigwig
Huh1_input_1.fq.gz Huh1_input_2.fq.gz Huh1_input.bigwig
input_PCSK9_mice_1.fq.gz input_PCSK9_mice_2.fq.gz PCSK9_mice_input.bigwig
siLuc1_H3K4me1_1.fq.gz siLuc1_H3K4me1_2.fq.gz siluc1_H3K4me1.bigwig
siLuc2_H3K4me1_1.fq.gz siLuc2_H3K4me1_2.fq.gz siluc2_H3K4me1.bigwig
siLuc3_H3K4me1_1.fq.gz siLuc3_H3K4me1_2.fq.gz siluc3_H3K4me1.bigwig
siLuc4_H3K4me1_1.fq.gz siLuc4_H3K4me1_2.fq.gz siluc4_H3K4me1.bigwig
sika1_H3K4me1_1.fq.gz sika1_H3K4me1_2.fq.gz sika1_H3K4me1.bigwig
sika2_H3K4me1_1.fq.gz sika2_H3K4me1_2.fq.gz sika2_H3K4me1.bigwig
sika3_H3K4me1_1.fq.gz sika3_H3K4me1_2.fq.gz sika3_H3K4me1.bigwig
sika4_H3K4me1_1.fq.gz sika4_H3K4me1_2.fq.gz sika4_H3K4me1.bigwig
siha1_H3K4me1_1.fq.gz siha1_H3K4me1_2.fq.gz siha1_H3K4me1.bigwig
siha2_H3K4me1_1.fq.gz siha2_H3K4me1_2.fq.gz siha2_H3K4me1.bigwig
siha3_H3K4me1_1.fq.gz siha3_H3K4me1_2.fq.gz siha3_H3K4me1.bigwig
siha4_H3K4me1_1.fq.gz siha4_H3K4me1_2.fq.gz siha4_H3K4me1.bigwig
siLuc1_H3K4me2_1.fq.gz siLuc1_H3K4me2_2.fq.gz siLuc1_H3K4me2.bigwig
siLuc2_H3K4me2_1.fq.gz siLuc2_H3K4me2_2.fq.gz siLuc2_H3K4me2.bigwig
siLuc3_H3K4me2_1.fq.gz siLuc3_H3K4me2_2.fq.gz siLuc3_H3K4me2.bigwig
siLuc4_H3K4me2_1.fq.gz siLuc4_H3K4me2_2.fq.gz siLuc4_H3K4me2.bigwig
sika1_H3K4me2_1.fq.gz sika1_H3K4me2_2.fq.gz sika1_H3K4me2.bigwig
sika2_H3K4me2_1.fq.gz sika2_H3K4me2_2.fq.gz sika2_H3K4me2.bigwig
sika3_H3K4me2_1.fq.gz sika3_H3K4me2_2.fq.gz sika3_H3K4me2.bigwig
sika4_H3K4me2_1.fq.gz sika4_H3K4me2_2.fq.gz sika4_H3K4me2.bigwig
siha1_H3K4me2_1.fq.gz siha1_H3K4me2_2.fq.gz siha1_H3K4me2.bigwig
siha2_H3K4me2_1.fq.gz siha2_H3K4me2_2.fq.gz siha2_H3K4me2.bigwig
siha3_H3K4me2_1.fq.gz siha3_H3K4me2_2.fq.gz siha3_H3K4me2.bigwig
siha4_H3K4me2_1.fq.gz siha4_H3K4me2_2.fq.gz siha4_H3K4me2.bigwig
siLuc1_H3K27ac_1.fq.gz siLuc1_H3K27ac_2.fq.gz siLuc1_H3K27ac.bigwig
siLuc2_H3K27ac_1.fq.gz siLuc2_H3K27ac_2.fq.gz siLuc2_H3K27ac.bigwig
siLuc3_H3K27ac_1.fq.gz siLuc3_H3K27ac_2.fq.gz siLuc3_H3K27ac.bigwig
siLuc4_H3K27ac_1.fq.gz siLuc4_H3K27ac_2.fq.gz siLuc4_H3K27ac.bigwig
sika1_H3K27ac_1.fq.gz sika1_H3K27ac_2.fq.gz sika1_H3K27ac.bigwig
sika2_H3K27ac_1.fq.gz sika2_H3K27ac_2.fq.gz sika2_H3K27ac.bigwig
sika3_H3K27ac_1.fq.gz sika3_H3K27ac_2.fq.gz sika3_H3K27ac.bigwig
sika4_H3K27ac_1.fq.gz sika4_H3K27ac_2.fq.gz sika4_H3K27ac.bigwig
siha1_H3K27ac_1.fq.gz siha1_H3K27ac_2.fq.gz siha1_H3K27ac.bigwig
siha2_H3K27ac_1.fq.gz siha2_H3K27ac_2.fq.gz siha2_H3K27ac.bigwig

siha3_H3K27ac_1.fq.gz siha3_H3K27ac_2.fq.gz siha3_H3K27ac.bigwig
siha4_H3K27ac_1.fq.gz siha4_H3K27ac_2.fq.gz siha4_H3K27ac.bigwig
siLuc1_H3K27me3_1.fq.gz siLuc1_H3K27me3_2.fq.gz siLuc1_H3K27me3.bigwig
siLuc2_H3K27me3_1.fq.gz siLuc2_H3K27me3_2.fq.gz siLuc2_H3K27me3.bigwig
siLuc3_H3K27me3_1.fq.gz siLuc3_H3K27me3_2.fq.gz siLuc3_H3K27me3.bigwig
siLuc4_H3K27me3_1.fq.gz siLuc4_H3K27me3_2.fq.gz siLuc4_H3K27me3.bigwig
sika1_H3K27me3_1.fq.gz sika1_H3K27me3_2.fq.gz sika1_H3K27me3.bigwig
sika2_H3K27me3_1.fq.gz sika2_H3K27me3_2.fq.gz sika2_H3K27me3.bigwig
sika3_H3K27me3_1.fq.gz sika3_H3K27me3_2.fq.gz sika3_H3K27me3.bigwig
sika4_H3K27me3_1.fq.gz sika4_H3K27me3_2.fq.gz sika4_H3K27me3.bigwig
siha1_H3K27me3_1.fq.gz siha1_H3K27me3_2.fq.gz siha1_H3K27me3.bigwig
siha2_H3K27me3_1.fq.gz siha2_H3K27me3_2.fq.gz siha2_H3K27me3.bigwig
siha3_H3K27me3_1.fq.gz siha3_H3K27me3_2.fq.gz siha3_H3K27me3.bigwig
siha4_H3K27me3_1.fq.gz siha4_H3K27me3_2.fq.gz siha4_H3K27me3.bigwig
flox1_me1_1.fq.gz flox1_me1_2.fq.gz flox1_me1.bigwig
flox2_me1_1.fq.gz flox2_me1_2.fq.gz flox2_me1.bigwig
flox3_me1_1.fq.gz flox3_me1_2.fq.gz flox3_me1.bigwig
flox1_me2_1.fq.gz flox1_me2_2.fq.gz flox1_me2.bigwig
flox2_me2_1.fq.gz flox2_me2_2.fq.gz flox2_me2.bigwig
flox3_me2_1.fq.gz flox3_me2_2.fq.gz flox3_me2.bigwig
flox1_ac_1.fq.gz flox1_ac_2.fq.gz flox1_ac.bigwig
flox2_ac_1.fq.gz flox2_ac_2.fq.gz flox2_ac.bigwig
flox3_ac_1.fq.gz flox3_ac_2.fq.gz flox3_ac.bigwig
flox1_me3_1.fq.gz flox1_me3_2.fq.gz flox1_me3.bigwig
flox2_me3_1.fq.gz flox2_me3_2.fq.gz flox2_me3.bigwig
flox3_me3_1.fq.gz flox3_me3_2.fq.gz flox3_me3.bigwig
LKO1_me1_1.fq.gz LKO1_me1_2.fq.gz LKO1_me1.bigwig
LKO2_me1_1.fq.gz LKO2_me1_2.fq.gz LKO2_me1.bigwig
LKO3_me1_1.fq.gz LKO3_me1_2.fq.gz LKO3_me1.bigwig
LKO1_me2_1.fq.gz LKO1_me2_2.fq.gz LKO1_me2.bigwig
LKO2_me2_1.fq.gz LKO2_me2_2.fq.gz LKO2_me2.bigwig
LKO3_me2_1.fq.gz LKO3_me2_2.fq.gz LKO3_me2.bigwig
LKO1_ac_1.fq.gz LKO1_ac_2.fq.gz LKO1_ac.bigwig
LKO2_ac_1.fq.gz LKO2_ac_2.fq.gz LKO2_ac.bigwig
LKO3_ac_1.fq.gz LKO3_ac_2.fq.gz LKO3_ac.bigwig
LKO1_me3_1.fq.gz LKO1_me3_2.fq.gz LKO1_me3.bigwig
LKO2_me3_1.fq.gz LKO2_me3_2.fq.gz LKO2_me3.bigwig
LKO3_me3_1.fq.gz LKO3_me3_2.fq.gz LKO3_me3.bigwig
input_1.fq.gz input_2.fq.gz hro_input.bigwig
input_mice_1.fq.gz input_mice_2.fq.gz mice_input.bigwig
NC1_CREBH_1.fq.gz NC1_CREBH_2.fq.gz NC1_CREBH.bigwig
NC2_CREBH_1.fq.gz NC2_CREBH_2.fq.gz NC2_CREBH.bigwig
NC3_CREBH_1.fq.gz NC3_CREBH_2.fq.gz NC3_CREBH.bigwig
KA1_CREBH_1.fq.gz KA1_CREBH_2.fq.gz KA1_CREBH.bigwig
KA2_CREBH_1.fq.gz KA2_CREBH_2.fq.gz KA2_CREBH.bigwig
KA3_CREBH_1.fq.gz KA3_CREBH_2.fq.gz KA3_CREBH.bigwig
WT1_antiCREBH_1.fq.gz WT1_antiCREBH_2.fq.gz WT1_antiCREBH.bigwig
WT2_antiCREBH_1.fq.gz WT2_antiCREBH_2.fq.gz WT2_antiCREBH.bigwig
WT3_antiCREBH_1.fq.gz WT3_antiCREBH_2.fq.gz WT3_antiCREBH.bigwig
WT4_antiCREBH_1.fq.gz WT4_antiCREBH_2.fq.gz WT4_antiCREBH.bigwig
LKO1_antiCREBH_1.fq.gz LKO1_antiCREBH_2.fq.gz LKO1_antiCREBH.bigwig
LKO2_antiCREBH_1.fq.gz LKO2_antiCREBH_2.fq.gz LKO2_antiCREBH.bigwig
LKO3_antiCREBH_1.fq.gz LKO3_antiCREBH_2.fq.gz LKO3_antiCREBH.bigwig
LKO4_antiCREBH_1.fq.gz LKO4_antiCREBH_2.fq.gz LKO4_antiCREBH.bigwig

Genome browser session
(e.g. UCSC)

https://www.ncbi.nlm.nih.gov/geo/query/acc.cgi?acc=GSE287736. Secure token of ChIP-seq Data(GSE287736) for reviewers: qpkpaceolxithix

Methodology

Replicates

ChIP-seq sample replicate(s) type
HROhep03_siLuc_H3K4me1 4 biological
HROhep03_siKDM6A_H3K4me1 4 biological
HROhep03_siHNF4A_H3K4me1 4 biological
HROhep03_siLuc_H3K4me2 4 biological
HROhep03_siKDM6A_H3K4me2 4 biological
HROhep03_siHNF4A_H3K4me2 4 biological
HROhep03_siLuc_H3K27ac 4 biological
HROhep03_siKDM6A_H3K27ac 4 biological
HROhep03_siHNF4A_H3K27ac 4 biological
HROhep03_siLuc_H3K27me3 4 biological
HROhep03_siKDM6A_H3K27me3 4 biological
HROhep03_siHNF4A_H3K27me3 4 biological
HROHep03_siLuc_CREBH 3 biological
HROHep03_siKDM6A_CREBH 3 biological

mice_flox_H3K4me1 3 biological
mice_flox_H3K4me2 3 biological
mice_flox_H3K27ac 3 biological
mice_flox_H3K27me3 3 biological
mice_LKO_H3K4me1 3 biological
mice_LKO_H3K4me2 3 biological
mice_LKO_H3K27ac 3 biological
mice_LKO_H3K27me3 3 biological
HROhep03_input 1 biological
Mice_input 1 biological
Huh1_siLuc_H3K4me1 4 biological
Huh1_siKDM6A_H3K4me1 4 biological
Huh1_siHNF4A_H3K4me1 4 biological
Huh1_siLuc_H3K4me2 4 biological
Huh1_siKDM6A_H3K4me2 4 biological
Huh1_siHNF4A_H3K4me2 4 biological
Huh1_siLuc_H3K27ac 4 biological
Huh1_siKDM6A_H3K27ac 4 biological
Huh1_siHNF4A_H3K27ac 4 biological
Huh1_siLuc_H3K27me3 4 biological
Huh1_siKDM6A_H3K27me3 4 biological
Huh1_siHNF4A_H3K27me3 4 biological
PCSK9_mice_WT_antiCREBH 4 biological
PCSK9_mice_LKO_antiCREBH 4 biological
Huh1_input 1 biological
PCSK9_mice_input 1 biological

Sequencing depth

ChIP-seq samples total reads unique reads length of the reads
HROhep03_siLuc_H3K4me1_rep1 39625952 27525826 150
HROhep03_siLuc_H3K4me1_rep2 36427559 25921671 150
HROhep03_siLuc_H3K4me1_rep3 32301736 22114948 150
HROhep03_siLuc_H3K4me1_rep4 28075953 19130727 150
HROhep03_siKDM6A_H3K4me1_rep1 38304538 27124766 150
HROhep03_siKDM6A_H3K4me1_rep2 34453562 24933104 150
HROhep03_siKDM6A_H3K4me1_rep3 30077445 20974858 150
HROhep03_siKDM6A_H3K4me1_rep4 26174347 18203324 150
HROhep03_siHNF4A_H3K4me1_rep1 77933617 55016306 150
HROhep03_siHNF4A_H3K4me1_rep2 71996002 51868322 150
HROhep03_siHNF4A_H3K4me1_rep3 64761619 45013613 150
HROhep03_siHNF4A_H3K4me1_rep4 52815986 36593799 150
HROhep03_siLuc_H3K4me2_rep1 80873920 53501162 150
HROhep03_siLuc_H3K4me2_rep2 74196132 50783963 150
HROhep03_siLuc_H3K4me2_rep3 68042698 44587317 150
HROhep03_siLuc_H3K4me2_rep4 56929525 37141880 150
HROhep03_siKDM6A_H3K4me2_rep1 63402875 42952220 150
HROhep03_siKDM6A_H3K4me2_rep2 58420583 40804511 150
HROhep03_siKDM6A_H3K4me2_rep3 51687639 34854228 150
HROhep03_siKDM6A_H3K4me2_rep4 41835504 28126115 150
HROhep03_siHNF4A_H3K4me2_rep1 75097746 50175880 150
HROhep03_siHNF4A_H3K4me2_rep2 66502240 45987031 150
HROhep03_siHNF4A_H3K4me2_rep3 57259598 38058426 150
HROhep03_siHNF4A_H3K4me2_rep4 51547767 34026241 150
HROhep03_siLuc_H3K27ac_rep1 65645873 44694332 150
HROhep03_siLuc_H3K27ac_rep2 64644498 45100757 150
HROhep03_siLuc_H3K27ac_rep3 60129498 40222708 150
HROhep03_siLuc_H3K27ac_rep4 52034474 34647601 150
HROhep03_siKDM6A_H3K27ac_rep1 71334966 49696413 150
HROhep03_siKDM6A_H3K27ac_rep2 63209983 45128802 150
HROhep03_siKDM6A_H3K27ac_rep3 54494321 37530364 150
HROhep03_siKDM6A_H3K27ac_rep4 45817797 31407114 150
HROhep03_siHNF4A_H3K27ac_rep1 43143961 29719005 150
HROhep03_siHNF4A_H3K27ac_rep2 38311321 27078565 150
HROhep03_siHNF4A_H3K27ac_rep3 34966760 23729121 150
HROhep03_siHNF4A_H3K27ac_rep4 29170888 19715668 150
HROhep03_siLuc_H3K27me3_rep1 67165572 41548340 150
HROhep03_siLuc_H3K27me3_rep2 59545507 38175106 150
HROhep03_siLuc_H3K27me3_rep3 52207844 31997358 150
HROhep03_siLuc_H3K27me3_rep4 45638776 27823325 150
HROhep03_siKDM6A_H3K27me3_rep1 53017398 34065666 150
HROhep03_siKDM6A_H3K27me3_rep2 48462252 32100277 150
HROhep03_siKDM6A_H3K27me3_rep3 42341107 26916945 150
HROhep03_siKDM6A_H3K27me3_rep4 37010956 23428614 150
HROhep03_siHNF4A_H3K27me3_rep1 62059450 39329755 150
HROhep03_siHNF4A_H3K27me3_rep2 56120527 36672986 150
HROhep03_siHNF4A_H3K27me3_rep3 49836677 31200641 150
HROhep03_siHNF4A_H3K27me3_rep4 42609248 26560133 150

mice_flox_H3K4me1_rep1 63575611 34363037 150
mice_flox_H3K4me1_rep2 59491770 32775147 150
mice_flox_H3K4me1_rep3 57872851 31502694 150
mice_flox_H3K4me2_rep1 66726387 37742678 150
mice_flox_H3K4me2_rep2 59633715 34202885 150
mice_flox_H3K4me2_rep3 55822997 31645752 150
mice_flox_H3K27ac_rep1 43494088 20343830 150
mice_flox_H3K27ac_rep2 39427605 19407225 150
mice_flox_H3K27ac_rep3 49793289 27426294 150
mice_flox_H3K27me3_rep1 33923189 14415289 150
mice_flox_H3K27me3_rep2 33001399 13680955 150
mice_flox_H3K27me3_rep3 49060224 23707280 150
mice_LKO_H3K4me1_rep1 58632169 31862062 150
mice_LKO_H3K4me1_rep2 71787122 37925219 150
mice_LKO_H3K4me1_rep3 41240188 22119934 150
mice_LKO_H3K4me2_rep1 56083143 30799210 150
mice_LKO_H3K4me2_rep2 55017717 30245568 150
mice_LKO_H3K4me2_rep3 57602357 31559818 150
mice_LKO_H3K27ac_rep1 31346005 16104880 150
mice_LKO_H3K27ac_rep2 83755678 45788350 150
mice_LKO_H3K27ac_rep3 60703463 31502324 150
mice_LKO_H3K27me3_rep1 41220446 18886676 150
mice_LKO_H3K27me3_rep2 75747916 39006971 150
mice_LKO_H3K27me3_rep3 34162873 15982042 150
HROhep03_input 55018582 26994059 150
Mice_input 37217113 10831223 150
Huh1_siLuc_H3K4me1_rep1 66285405 35879642 150
Huh1_siLuc_H3K4me1_rep2 66223617 36380097 150
Huh1_siLuc_H3K4me1_rep3 64587903 35024430 150
Huh1_siLuc_H3K4me1_rep4 52350783 28539074 150
Huh1_siKDM6A_H3K4me1_rep1 61356755 34769417 150
Huh1_siKDM6A_H3K4me1_rep2 58638017 32953051 150
Huh1_siKDM6A_H3K4me1_rep3 55827771 30910846 150
Huh1_siKDM6A_H3K4me1_rep4 55896655 31136233 150
Huh1_siHNF4A_H3K4me1_rep1 44743073 27376382 150
Huh1_siHNF4A_H3K4me1_rep2 49256194 29809908 150
Huh1_siHNF4A_H3K4me1_rep3 48885075 29024606 150
Huh1_siHNF4A_H3K4me1_rep4 49287042 29320745 150
Huh1_siLuc_H3K4me2_rep1 45793729 25027421 150
Huh1_siLuc_H3K4me2_rep2 53985927 29584611 150
Huh1_siLuc_H3K4me2_rep3 52180774 27825427 150
Huh1_siLuc_H3K4me2_rep4 52590197 27998015 150
Huh1_siKDM6A_H3K4me2_rep1 55450309 32240098 150
Huh1_siKDM6A_H3K4me2_rep2 59207538 34298936 150
Huh1_siKDM6A_H3K4me2_rep3 56607566 32226045 150
Huh1_siKDM6A_H3K4me2_rep4 56193222 32316504 150
Huh1_siHNF4A_H3K4me2_rep1 58400244 33573544 150
Huh1_siHNF4A_H3K4me2_rep2 59514594 33982316 150
Huh1_siHNF4A_H3K4me2_rep3 52068749 29269456 150
Huh1_siHNF4A_H3K4me2_rep4 54810534 31052909 150
Huh1_siLuc_H3K27ac_rep1 56925683 33801014 150
Huh1_siLuc_H3K27ac_rep2 60250209 35378056 150
Huh1_siLuc_H3K27ac_rep3 59670800 34501885 150
Huh1_siLuc_H3K27ac_rep4 53837197 31390893 150
Huh1_siKDM6A_H3K27ac_rep1 61031034 36421652 150
Huh1_siKDM6A_H3K27ac_rep2 66968573 39743460 150
Huh1_siKDM6A_H3K27ac_rep3 61690246 36130350 150
Huh1_siKDM6A_H3K27ac_rep4 54856355 32354296 150
Huh1_siHNF4A_H3K27ac_rep1 60933725 37814616 150
Huh1_siHNF4A_H3K27ac_rep2 58165608 35819335 150
Huh1_siHNF4A_H3K27ac_rep3 61670711 37473411 150
Huh1_siHNF4A_H3K27ac_rep4 53589800 32795371 150
Huh1_siLuc_H3K27me3_rep1 45149015 17880796 150
Huh1_siLuc_H3K27me3_rep2 45758673 17861090 150
Huh1_siLuc_H3K27me3_rep3 45572839 17496195 150
Huh1_siLuc_H3K27me3_rep4 51838478 19893387 150
Huh1_siKDM6A_H3K27me3_rep1 40474704 16620186 150
Huh1_siKDM6A_H3K27me3_rep2 50893271 20552310 150
Huh1_siKDM6A_H3K27me3_rep3 44421627 17677165 150
Huh1_siKDM6A_H3K27me3_rep4 48647866 19359317 150
Huh1_siHNF4A_H3K27me3_rep1 41600310 17053218 150
Huh1_siHNF4A_H3K27me3_rep2 50696768 20421523 150
Huh1_siHNF4A_H3K27me3_rep3 42303895 16787291 150
Huh1_siHNF4A_H3K27me3_rep4 47256069 18773044 150
Huh1_input 53016012 16366413 150
PCSK9_mice_input 97187372 29755599 150

HROHep03_siLuc_CREBH_rep1 68473992 66944016 150
HROHep03_siLuc_CREBH_rep2 66988915 61543355 150
HROHep03_siLuc_CREBH_rep3 55928508 51574547 150
HROHep03_siKDM6A_CREBH_rep1 62585938 61030111 150
HROHep03_siKDM6A_CREBH_rep2 49005541 46327229 150
HROHep03_siKDM6A_CREBH_rep3 59423287 54027453 150
PCSK9_mice_WT_CREBH_rep1 51533190 41154682 150
PCSK9_mice_WT_CREBH_rep2 51718309 41201075 150
PCSK9_mice_WT_CREBH_rep3 48870200 39311486 150
PCSK9_mice_WT_CREBH_rep4 81596057 68803909 150
PCSK9_mice_LKO_CREBH_rep1 48736125 37513282 150
PCSK9_mice_LKO_CREBH_rep2 48036933 36844338 150
PCSK9_mice_LKO_CREBH_rep3 49106978 38007539 150
PCSK9_mice_LKO_CREBH_rep4 93605109 73444005 150

Antibodies

control rabbit IgG (sc-2027, Santa Cruz), anti-H3K27ac (ab177178, Abcam), anti-H3K4me1 (ab8895, Abcam), anti-H3K4me2 (ab7766, Abcam), anti-H3K27me3 (07-449,Millipore) and anti-CREBH (HPA040671, Sigma Aldrich)

Peak calling parameters

Sequenced raw data (in fastq) were aligned to human hg38 or mouse mm10 genome using Bowtie2 program with all default settings.
All peaks were determined by the HOMER findPeaks program against the input samples with the following options:
-i <input tag directory> (input sample)
-tbp 1 (maximum 1 tag per bp to count);
-inputtbp 1 (maximum 1 tag per bp to count in Input);
other default settings:
-gsize (Set effective mappable genome size, default: 2e9)
-F (fold enrichment over input tag count, default: 4.0);
-L (fold enrichment over local tag count, default: 4.0);
-C (fold enrichment limit of expected unique tag positions, default: 2.0);
-fdr (False discovery rate, default = 0.001)

Data quality

ChIP-seq samples peak number
HROhep03_siLuc_H3K4me1_rep1 101396
HROhep03_siLuc_H3K4me1_rep2 109511
HROhep03_siLuc_H3K4me1_rep3 99619
HROhep03_siLuc_H3K4me1_rep4 90705
HROhep03_siKDM6A_H3K4me1_rep1 95365
HROhep03_siKDM6A_H3K4me1_rep2 101322
HROhep03_siKDM6A_H3K4me1_rep3 92376
HROhep03_siKDM6A_H3K4me1_rep4 83434
HROhep03_siHNF4A_H3K4me1_rep1 125052
HROhep03_siHNF4A_H3K4me1_rep2 119211
HROhep03_siHNF4A_H3K4me1_rep3 122319
HROhep03_siHNF4A_H3K4me1_rep4 104392
HROhep03_siLuc_H3K4me2_rep1 74885
HROhep03_siLuc_H3K4me2_rep2 72696
HROhep03_siLuc_H3K4me2_rep3 72586
HROhep03_siLuc_H3K4me2_rep4 67243
HROhep03_siKDM6A_H3K4me2_rep1 68798
HROhep03_siKDM6A_H3K4me2_rep2 67267
HROhep03_siKDM6A_H3K4me2_rep3 61909
HROhep03_siKDM6A_H3K4me2_rep4 60470
HROhep03_siHNF4A_H3K4me2_rep1 78100
HROhep03_siHNF4A_H3K4me2_rep2 78518
HROhep03_siHNF4A_H3K4me2_rep3 73051
HROhep03_siHNF4A_H3K4me2_rep4 75094
HROhep03_siLuc_H3K27ac_rep1 74318
HROhep03_siLuc_H3K27ac_rep2 73394
HROhep03_siLuc_H3K27ac_rep3 70934
HROhep03_siLuc_H3K27ac_rep4 72031
HROhep03_siKDM6A_H3K27ac_rep1 69572
HROhep03_siKDM6A_H3K27ac_rep2 71283
HROhep03_siKDM6A_H3K27ac_rep3 65196
HROhep03_siKDM6A_H3K27ac_rep4 65570
HROhep03_siHNF4A_H3K27ac_rep1 64453
HROhep03_siHNF4A_H3K27ac_rep2 61366
HROhep03_siHNF4A_H3K27ac_rep3 65914
HROhep03_siHNF4A_H3K27ac_rep4 59940
HROhep03_siLuc_H3K27me3_rep1 80575
HROhep03_siLuc_H3K27me3_rep2 70505
HROhep03_siLuc_H3K27me3_rep3 78781
HROhep03_siLuc_H3K27me3_rep4 64816
HROhep03_siKDM6A_H3K27me3_rep1 84168
HROhep03_siKDM6A_H3K27me3_rep2 77468
HROhep03_siKDM6A_H3K27me3_rep3 66867
HROhep03_siKDM6A_H3K27me3_rep4 75659
HROhep03_siHNF4A_H3K27me3_rep1 98659

HROhep03_siHNF4A_H3K27me3_rep2 90897
HROhep03_siHNF4A_H3K27me3_rep3 80190
HROhep03_siHNF4A_H3K27me3_rep4 88941
mice_flox_H3K4me1_rep1 21175
mice_flox_H3K4me1_rep2 21215
mice_flox_H3K4me1_rep3 20930
mice_flox_H3K4me2_rep1 28429
mice_flox_H3K4me2_rep2 28831
mice_flox_H3K4me2_rep3 28989
mice_flox_H3K27ac_rep1 29641
mice_flox_H3K27ac_rep2 26781
mice_flox_H3K27ac_rep3 29309
mice_flox_H3K27me3_rep1 10515
mice_flox_H3K27me3_rep2 12375
mice_flox_H3K27me3_rep3 5563
mice_LKO_H3K4me1_rep1 5280
mice_LKO_H3K4me1_rep2 16523
mice_LKO_H3K4me1_rep3 20414
mice_LKO_H3K4me2_rep1 31374
mice_LKO_H3K4me2_rep2 31974
mice_LKO_H3K4me2_rep3 31592
mice_LKO_H3K27ac_rep1 28902
mice_LKO_H3K27ac_rep2 24878
mice_LKO_H3K27ac_rep3 24121
mice_LKO_H3K27me3_rep1 9295
mice_LKO_H3K27me3_rep2 1237
mice_LKO_H3K27me3_rep3 8299
PCSK9_mice_WT_antiCREBH_rep1 696
PCSK9_mice_WT_antiCREBH_rep2 679
PCSK9_mice_WT_antiCREBH_rep3 620
PCSK9_mice_WT_antiCREBH_rep4 1591
PCSK9_mice_LKO_antiCREBH_rep1 154
PCSK9_mice_LKO_antiCREBH_rep2 133
PCSK9_mice_LKO_antiCREBH_rep3 172
PCSK9_mice_LKO_antiCREBH_rep4 543
Huh1_siLuc_H3K4me1_rep1 128707
Huh1_siLuc_H3K4me1_rep2 105393
Huh1_siLuc_H3K4me1_rep3 127991
Huh1_siLuc_H3K4me1_rep4 134058
Huh1_siKDM6A_H3K4me1_rep1 110432
Huh1_siKDM6A_H3K4me1_rep2 106789
Huh1_siKDM6A_H3K4me1_rep3 101251
Huh1_siKDM6A_H3K4me1_rep4 101960
Huh1_siHNF4A_H3K4me1_rep1 131113
Huh1_siHNF4A_H3K4me1_rep2 137823
Huh1_siHNF4A_H3K4me1_rep3 135841
Huh1_siHNF4A_H3K4me1_rep4 135840
Huh1_siLuc_H3K4me2_rep1 90045
Huh1_siLuc_H3K4me2_rep2 96581
Huh1_siLuc_H3K4me2_rep3 94056
Huh1_siLuc_H3K4me2_rep4 94374
Huh1_siKDM6A_H3K4me2_rep1 78808
Huh1_siKDM6A_H3K4me2_rep2 80824
Huh1_siKDM6A_H3K4me2_rep3 78690
Huh1_siKDM6A_H3K4me2_rep4 79208
Huh1_siHNF4A_H3K4me2_rep1 83385
Huh1_siHNF4A_H3K4me2_rep2 83865
Huh1_siHNF4A_H3K4me2_rep3 96264
Huh1_siHNF4A_H3K4me2_rep4 79836
Huh1_siLuc_H3K27ac_rep1 60915
Huh1_siLuc_H3K27ac_rep2 63234
Huh1_siLuc_H3K27ac_rep3 62599
Huh1_siLuc_H3K27ac_rep4 70552
Huh1_siKDM6A_H3K27ac_rep1 60087
Huh1_siKDM6A_H3K27ac_rep2 63234
Huh1_siKDM6A_H3K27ac_rep3 62599
Huh1_siKDM6A_H3K27ac_rep4 55674
Huh1_siHNF4A_H3K27ac_rep1 58694
Huh1_siHNF4A_H3K27ac_rep2 64403
Huh1_siHNF4A_H3K27ac_rep3 58396
Huh1_siHNF4A_H3K27ac_rep4 60741
Huh1_siLuc_H3K27me3_rep1 78178
Huh1_siLuc_H3K27me3_rep2 78622
Huh1_siLuc_H3K27me3_rep3 77281
Huh1_siLuc_H3K27me3_rep4 85201
Huh1_siKDM6A_H3K27me3_rep1 74961

Huh1_siKDM6A_H3K27me3_rep2 88050
Huh1_siKDM6A_H3K27me3_rep3 79123
Huh1_siKDM6A_H3K27me3_rep4 84407
Huh1_siHNF4A_H3K27me3_rep1 67117
Huh1_siHNF4A_H3K27me3_rep2 78269
Huh1_siHNF4A_H3K27me3_rep3 65529
Huh1_siHNF4A_H3K27me3_rep4 72837
HROHep03_siLuc_CREBH_rep1 8142
HROHep03_siLuc_CREBH_rep2 40506
HROHep03_siLuc_CREBH_rep3 44047
HROHep03_siKDM6A_CREBH_rep1 2504
HROHep03_siKDM6A_CREBH_rep2 29849
HROHep03_siKDM6A_CREBH_rep3 46747

Software

Homer(v5.1); Bowtie2 (v2.5.4); MACS2 (v2.2.9.1)

Flow Cytometry

Plots

Confirm that:

☐ The axis labels state the marker and fluorochrome used (e.g. CD4-FITC).

☐ The axis scales are clearly visible. Include numbers along axes only for bottom left plot of group (a 'group' is an analysis of identical markers).

☐ All plots are contour plots with outliers or pseudocolor plots.

☐ A numerical value for number of cells or percentage (with statistics) is provided.

Methodology

Sample preparation

Describe the sample preparation, detailing the biological source of the cells and any tissue processing steps used.

Instrument

Identify the instrument used for data collection, specifying make and model number.

Software

Describe the software used to collect and analyze the flow cytometry data. For custom code that has been deposited into a community repository, provide accession details.

Cell population abundance

Describe the abundance of the relevant cell populations within post-sort fractions, providing details on the purity of the samples and how it was determined.

Gating strategy

Describe the gating strategy used for all relevant experiments, specifying the preliminary FSC/SSC gates of the starting cell population, indicating where boundaries between "positive" and "negative" staining cell populations are defined.

☐ Tick this box to confirm that a figure exemplifying the gating strategy is provided in the Supplementary Information.

Magnetic resonance imaging

Experimental design

Design type

Indicate task or resting state; event-related or block design.

Design specifications

Specify the number of blocks, trials or experimental units per session and/or subject, and specify the length of each trial or block (if trials are blocked) and interval between trials.

Behavioral performance measures

State number and/or type of variables recorded (e.g. correct button press, response time) and what statistics were used to establish that the subjects were performing the task as expected (e.g. mean, range, and/or standard deviation across subjects).

Acquisition

Imaging type(s)

Specify: functional, structural, diffusion, perfusion.

Field strength

Specify in Tesla

Sequence & imaging parameters

Specify the pulse sequence type (gradient echo, spin echo, etc.), imaging type (EPI, spiral, etc.), field of view, matrix size, slice thickness, orientation and TE/TR/flip angle.

Area of acquisition

State whether a whole brain scan was used OR define the area of acquisition, describing how the region was determined.

Diffusion MRI ☐ Used ☐ Not used

Preprocessing

Preprocessing software	*Provide detail on software version and revision number and on specific parameters (model/functions, brain extraction, segmentation, smoothing kernel size, etc.).*
Normalization	*If data were normalized/standardized, describe the approach(es): specify linear or non-linear and define image types used for transformation OR indicate that data were not normalized and explain rationale for lack of normalization.*
Normalization template	*Describe the template used for normalization/transformation, specifying subject space or group standardized space (e.g. original Talairach, MNI305, ICBM152) OR indicate that the data were not normalized.*
Noise and artifact removal	*Describe your procedure(s) for artifact and structured noise removal, specifying motion parameters, tissue signals and physiological signals (heart rate, respiration).*
Volume censoring	*Define your software and/or method and criteria for volume censoring, and state the extent of such censoring.*

Statistical modeling & inference

Model type and settings	*Specify type (mass univariate, multivariate, RSA, predictive, etc.) and describe essential details of the model at the first and second levels (e.g. fixed, random or mixed effects; drift or auto-correlation).*
Effect(s) tested	*Define precise effect in terms of the task or stimulus conditions instead of psychological concepts and indicate whether ANOVA or factorial designs were used.*

Specify type of analysis: ☐ Whole brain ☐ ROI-based ☐ Both

Statistic type for inference (See Eklund et al. 2016)	*Specify voxel-wise or cluster-wise and report all relevant parameters for cluster-wise methods.*
Correction	*Describe the type of correction and how it is obtained for multiple comparisons (e.g. FWE, FDR, permutation or Monte Carlo).*

Models & analysis

n/a	Involved in the study
☐	☐ Functional and/or effective connectivity
☐	☐ Graph analysis
☐	☐ Multivariate modeling or predictive analysis

Functional and/or effective connectivity	*Report the measures of dependence used and the model details (e.g. Pearson correlation, partial correlation, mutual information).*
Graph analysis	*Report the dependent variable and connectivity measure, specifying weighted graph or binarized graph, subject- or group-level, and the global and/or node summaries used (e.g. clustering coefficient, efficiency, etc.).*
Multivariate modeling and predictive analysis	*Specify independent variables, features extraction and dimension reduction, model, training and evaluation metrics.*
